# From Nursing Homes to Geriatric Psychiatry: Contextual Factors Associated With the Admission of People With Dementia and Behaviour That Challenges—An Integrative Review

**DOI:** 10.1002/nop2.70592

**Published:** 2026-05-16

**Authors:** Imane Henni Rached, Ivonne Ledtermann, Renate Stemmer

**Affiliations:** ^1^ University of Bremen Bremen Germany; ^2^ Rheinhessen‐Fachklinik Alzey Alzey Germany; ^3^ Catholic University of Applied Sciences Mainz Mainz Germany

**Keywords:** admission, behavioural and psychological symptoms of dementia (BPSD), challenging behaviour, hospitalisation, interprofessional collaboration, nursing homes, people with dementia, psychiatry

## Abstract

**Aim:**

To systematically identify the contextual factors leading to (re)admission to psychiatry of people with dementia and behaviour that challenges living in nursing homes. Behaviour that challenges is understood as a social construct that reflects individual and environmental factors and their interaction.

**Design:**

The 5‐step Whittemore & Knafl method was followed for this integrative review.

**Data Sources:**

The databases MEDLINE, CINAHL, Cochrane Library, GeroLit and PsychINFO and the study registers ISRCTN, PROSPERO and DRKS were searched. Hand search and citation tracking were used.

**Methods:**

Inclusion and exclusion criteria were applied. Two independent reviewers assessed the quality of the studies via the Mixed Method Appraisal Tool and Joanna Briggs Institute's instruments. Data synthesis and integration were based on The Convergent Integrated Approach for Mixed Methods Systematic Reviews and the thematic analysis of Braun and Clarke.

**Results:**

1161 articles were identified, 8 articles/studies were retained. The contextual factors could be specified as ‘crisis determining factors’ (main theme). Three sub‐themes were identified: multifactoriality and ‐dimensionality of dementia and behaviour that challenges; structural and systemic barriers and lack of effective interprofessional and patient‐centred collaboration.

**Conclusion:**

Dementia crises often lead to hospitalisation. A key crisis‐determining factor is interprofessional collaboration. High‐quality studies on this topic are lacking. The existing results originate mainly from Europe and should be interpreted with caution in view of contextual and methodological limitations. Policymakers should promote cross‐sector care models, the deployment of psychiatric crisis teams, Advance Care Planning and digital communication tools.

**Reporting Method:**

We based our reporting on the PRISMA 2020 statement.

**Patient or Public Contribution:**

No patient or public contribution.

**Trial Registration:**

PROSPERO 2022 CRD42022367169; https://www.crd.york.ac.uk/prospero/display_record.php?ID=CRD42022367169

**Abbreviations**
ACP: Advance Care Planning
BGB: German Civil Code
BPSD: Behavioural and Psychological Symptoms of Dementia
DCSD: Dementia Care Sensitive Demands
DSCU: Demenita Special Care Unit
GP: General Practitioner
JBI: Joanna Briggs Institute
MMAT: Mixed Methods Appraisal Tool
MMSR: Mixed Methods Systematic Review
PRISMA: Preferred Reporting Items for Systematic Reviews and Meta‐Analyses
PsychKG: Mental Health Act (Germany)
StäB: inpatient‐equivalent home treatment

## 1 Introduction

Ninety percent of people with dementia develop Behavioural and Psychological Symptoms of Dementia (BPSD) during their illness, such as disturbances in motor function, perception, emotional experiences and day‐night rhythms. This results in stress for patients and carers, hospitalisation and inappropriate use of medications (Cerejeira et al. 2012). The cause of BPSD is still insufficiently understood, but there are indications that psychological, social and biological factors play important roles in their interaction (Cerejeira et al. 2012). BPSD increase the risk of being placed in a nursing home (Challis et al. 2017). Over 60% of people with dementia living in nursing homes have BPSD, which is a major care problem there (Majic et al. 2009). People with dementia are most frequently admitted to geriatric psychiatry due to states of confusion (31.9%), hallucinations/delusions (21.6%), aggressive behaviour (17.7%), depression (17.6%), refusal of food or fluids (14.4%), agitation (13.9%), suicidal behaviour (13.3%) and misbehaviour (13.0%). In 81.8% of the cases, self‐endangering or other‐endangering behaviour was the main reason for admission; in nursing homes, the main causes were aggression, agitation and misbehaviour (Wetterling et al. 2008). Sometimes there is a discrepancy between the aggressive, agitated behaviour reported by nursing home staff as a reason for the admission and the calm, cooperative behaviour of patients during the psychiatric admission process. Emergency admissions are most frequently due to agitation and aggression (Nejtek et al. 2011). The triggers of agitation and aggression in people with dementia are poorly understood. There are indications that the most aggressive escalations are related to interpersonal interactions (Whall et al. 2008).

## 2 Background

Some studies suggest that the primary reason for hospitalisation is BPSD (Caffrey 2010; Nejtek et al. 2011; Ouslander et al. 2010). In addition to BPSD there are further dementia‐specific factors (e.g., declining ability to communicate about symptoms or accidents) and context‐specific factors (e.g., care home‐specific factors, protection from legal consequences, qualifications of nursing staff, inadequate communication), which can lead to hospital admissions (Pohontsch et al. 2017).

There are many causes of BPSD, and it is not solely due to the disease ‘dementia’. We understand behaviour not only as a symptom of illness, but as a socially constructed result of individual factors (e.g., cognitive impairments, health problems, communication) and environmental factors (e.g., staffing, relationship quality, environmental design). A well‐founded assessment and intervention must include both levels and their interaction (Royal College of Psychiatrists et al. 2007). Therefore, we use the term ‘behaviour that challenges’ to emphasise the shared responsibility of the environment.

The processes, personal factors of the staff, deficient handling of behaviour that challenges, environmental factors and framework conditions in nursing homes are insufficiently reported. Studies are not specifically concerned with geriatric psychiatry but often focus on admissions to general hospitals (Fassmer et al. 2020; Matsuoka et al. 2019; Pohontsch et al. 2017). Although there are certainly parallels in treatment between psychiatry and general hospitals, the backgrounds leading to admission to geriatric psychiatry are probably different. Contextual information on the interface between nursing homes and geriatric psychiatry and the actors involved is lacking, so further research is needed.

A preliminary, rough search in MEDLINE, CINAHL and PROSPERO found no results that exactly investigated the phenomenon of interest. This integrative review used a comprehensive search that included all study designs and grey literature. The researchers chose an integrative review because it summarises literature, highlights research gaps and integrates various data sources.

## 3 Aim

The aim of this review is to systematically identify and analyse contextual factors associated with the (re)admission of people with dementia and behaviour that challenges to geriatric psychiatry. Contextual factors are defined as individual, environmental and systemic influences that are relevant to this process. The interface between geriatric psychiatry and nursing homes is focused on to uncover gaps and align future measures with actual needs.

### 3.1 Review Question

The research question is:

‘What contextual factors are associated with the (re)admission of people with dementia and behaviour that challenges from nursing homes to geriatric psychiatry?’

## 4 Methods

### 4.1 Design

The integrative review method was chosen to enable a comprehensive synthesis of the literature, considering a wide range of different study designs and methods. By integrating various data sources, deeper insights into the phenomenon under investigation can be obtained to systematically generate a knowledge base for nursing practice (Whittemore and Knafl 2005).

The integrative review approach according to Whittemore and Knafl (2005) formed the methodological framework and comprises five phases: problem identification, literature search, data evaluation, data analysis and presentation of results. The JBI method for Mixed Methods Systematic Review (MMSR), the *convergent integrated approach* (Lizarondo et al. 2020), was specifically integrated into the fourth step (data analysis) to systematically integrate qualitative and quantitative data. The combination of both models was thus sequential and complementary. We reported the review according to the Preferred Reporting Items for Systematic Review and Meta‐Analyses (PRISMA) guidelines (Page et al. 2021).

People with dementia are often admitted to geriatric psychiatry on the grounds that their behaviour is challenging. The reasons for hospitalisation are thus individualised (Hathaway et al. 2021; Matsuoka et al. 2019; Wetterling et al. 2008). This does not consider the fact that structural factors, for example, contextual and environmental factors, often promote hospitalisation (Pohontsch et al. 2017). The identification of contextual factors is necessary because little is known about their influence on admission processes in geriatric psychiatry. A systematic synthesis of existing studies from an international perspective can inform interface actors and decision‐makers about relevant determinants and enable the development of complex interventions for better care of people with dementia.

### 4.2 Search Methods

The comprehensive search strategy, according to a sensitive search principle, was based on the 10 steps for systematic literature research by Nordhausen and Hirt (2020) (1. search principle; 2. search components; 3. databases; 4. keywords; 5. catch words; 6./7. developing/checking search string; 8. search; 9. documenting, saving and exporting the search; 10. additional search options). The search components ‘dementia’, ‘challenging behaviour’, ‘(re)admission’ and ‘psychiatry’ were included in the review. ‘Contextual factors’ were not included as an additional search component, as they were not sufficiently operationalisable. The contextual factors were identified by analysing the studies/articles found. The term ‘nursing home’ was excluded from the final search strategy because initial test searches (reconstructed on 3 June 2025) showed that including the term excluded relevant results (see Supporting Information, Table S2).

We carried out a literature search from 2/2023 to 8/2023 and conducted an updated run of all the search methods on 15th October 2024. Five databases were searched: MEDLINE, CINAHL, the Cochrane Library, GeroLit, and PsychINFO. The search strategies used for the various databases, based on the search protocol provided by Hirt and Nordhausen (2022) are shown in Supporting Information, Table S1.

Additional search methods were used and carried out independently by two reviewers and discrepancies were resolved by discussion. Citation tracking was performed. The forward citation search was carried out via Google Scholar; the backwards citation search was carried out via manual review of the bibliographies. In addition, a manual search was conducted in the following three thematically relevant journals not indexed in the searched databases: Psychiatrische Pflege (focus: psychiatric nursing, issues 2016–2024, issues have only been archived online since 2016); Psychiatrische Pflege Heute (focus: psychiatric nursing, issues 2013–2024); and Journal für Qualitative Forschung in Pflege‐ und Gesundheitswissenschaft (focus: qualitative research in the care and health sector, issues 2014–2024, issues have only been archived online since 2014). Furthermore, the study registers ISRCTN, PROSPERO and DRKS (German Register of Clinical Studies) were searched (registration period 2013–2024), and the study authors of relevant hits were contacted.

The following filters were applied: Humans, published 2013–2023/2023‐15th October 2024 (updated search), German and English languages, abstracts and age limit (> 60).

### 4.3 Inclusion Criteria

The following a priori *inclusion criteria* were defined:

- Population: Older people with dementia (> 60 years) living in nursing homes or other long‐term care facilities who are admitted to (geronto‐)psychiatry owing to behaviour that challenges
- Study design: All study designs and expert opinions
- Setting/context: Interface between a nursing home and (geronto‐)psychiatry
- Publication period: 01/01/2013 to 15/10/2024
- Language: German or English

### 4.4 Screening

The articles found were transferred to Rayyan for Systematic Reviews. After removing duplicates, two reviewers independently checked the titles and abstracts for relevance and sorted all the clearly irrelevant papers. If the result was not clearly irrelevant, the full text was downloaded. Then the two reviewers independently assessed the eligibility of the studies based on the predefined inclusion criteria. Disagreements were resolved by discussion. Studies/articles were excluded if they included the wrong population (no dementia, no behaviour that challenges), the wrong phenomenon of interest (no admission, no contextual factors), or the wrong context (no admission to psychiatry, not from a nursing home).

Three studies/articles by the same authors were considered. Before these could be assessed, it had to be clarified to what extent they were based on different findings. After clarification by contacting the authors, the three sources could be considered and appraised separately (preliminary study (Pöschel et al. 2018), main study (Pöschel and Spannhorst 2018a), article with a different focus (Pöschel and Spannhorst 2018b)).

### 4.5 Assignment of Evidence Level and Quality Appraisal

The studies were evaluated based on their design using the appropriate JBI evidence hierarchy (Joanna Briggs Institute 2013, 2014) to assess the strength of the evidence. Studies with mixed methods were classified according to their components (quantitative, qualitative), and each statement was given a quality appraisal score. Statements from the overall results of the studies with mixed methods were given an overall rating based on the weakest individual component to avoid overrating (see Supporting Information, Tables S3–S5). Two independent reviewers assessed the methodological quality of the studies. Missing data was supplemented by contacting the authors where necessary. Discrepancies were clarified in discussions or with a third reviewer. Due to the heterogeneous study designs, several assessment tools were used. The reviewers assessed the studies/articles via the Mixed Method Appraisal Tool (MMAT) (Hong et al. 2018) and selected critical appraisal tools from the JBI (Checklist for Systematic Reviews and Research Syntheses (Aromataris et al. 2015), Checklist for Textual Evidence: Expert Opinion (McArthur et al. 2025)). The requirements of the specific tool were strictly adhered to. No studies were excluded based on the quality assessment.

### 4.6 Data Transformation and Extraction

Two researchers conducted the data extraction independently to ensure the objectivity and completeness of the data. The extracted results were then compared and checked for completeness. In line with the convergent integrated approach, the quantitative data was then jointly transformed into qualitative categories (qualitising) by converting into textual descriptions or narrative interpretations (Lizarondo et al. 2020). The final, transformed data was then transferred for each study into the ‘JBI Mixed Methods Data Extraction Form following a Convergent Integrated Approach’ (Aromataris et al. 2024). All statements from the included studies/articles are documented in Tables S3–S5 in the Supporting Information with corresponding page numbers and origin. The quotations from German‐language sources were translated into English using DeepL Pro. Quantitative results and their transformation into qualitatively interpreted data (‘qualitised data’), qualitative findings and aggregated, interpreted or opinion‐based statements were listed separately.

### 4.7 Data Synthesis and Integration

The extracted data were first coded independently by the two researchers to minimise subjective influence during the coding phase. After that the codes were discussed jointly by both researchers and merged in a reflective negotiation process. Categories were then formed by grouping and clustering related codes. These categories were visually grouped on pinboards to identify initial thematic clusters. These were iteratively reviewed, revised and compared with the extracted statements to ensure clear content coherence and selectivity.

The analysis followed the reflective approach of Braun and Clarke (2006, 2019) which emphasises the active role of the researchers. The calculation of inter‐coder reliability was deliberately omitted, as reflexive thematic analysis does not assume objective agreement, but rather that different perspectives and interpretations by researchers enrich the analysis and lead to a deeper understanding. The categories were formed as part of an iterative coordination process within the team, during which codes were jointly reviewed, discussed and consolidated on the basis of consensus. A coding table with the theme, sub‐themes, categories, codes, key quotes and the respective sources is presented in Table S6 in the Supporting Information. The results were presented narratively according to the sub‐themes and categories developed. Where possible, studies with high evidence were used as the primary basis, supplemented by weaker evidence for contextualisation or to broaden perspectives. Contributions with lower evidence strength were used in part to introduce topics that were subsequently supported by stronger evidence. This allowed low‐threshold sources to contribute without distorting the significance of the findings.

### 4.8 Deviations From the Protocol

The a priori protocol was registered in PROSPERO. The following deviations occurred:

- The search term ‘nursing home’ was omitted because test searches yielded mostly irrelevant hits (e.g., nursing home admission) and significantly limited the number of hits. Instead, nursing home affiliation was identified at the full‐text level. A reconstructed test search from 3 June 2025 supports this decision (see Supporting Information, Table S2).
- Inclusion and exclusion criteria: The age limit was set at over 60 years, as older people are typically treated in geriatric psychiatric facilities from this age onwards. However, this may limit the generalisability of the results to younger people.
- Quality appraisal: The included studies were primarily assessed using the MMAT (Hong et al. 2018), as this tool takes into account the evaluation of different designs of primary studies. Studies that could not be assessed using the MMAT (e.g., opinion papers, systematic reviews) were rated using specific JBI checklists (Aromataris et al. 2015; McArthur et al. 2025). To ensure methodological consistency and a more uniform assessment framework, the use of AMSTAR was avoided.
- Data synthesis: The data synthesis was not carried out exclusively in a narrative manner, as originally planned, but was based on the thematic analysis described by Braun and Clarke (Braun and Clarke 2006), as a purely narrative summary of the heterogeneous data set was unable to adequately capture key patterns.

### 4.9 Dealing With Reporting Biases and Assessing the Reliability of the Results

We have taken potential publication and reporting biases into account when interpreting the results by critically assessing the quality of the studies and the extracted findings (JBI Quality Appraisal Tools and JBI Evidence hierarchies), the heterogeneity of study designs, and the consistency of findings. We assess the certainty of the results as limited to low, as the data synthesis included not only a small number of empirical studies but also opinion papers, as well as indirect findings and interpretative conclusions, which increases susceptibility to bias and limits the generalisability and robustness of the evidence. This highlights that the evidence base has a significant research gap.

## 5 Results

### 5.1 Study Selection

**FIGURE 1:**
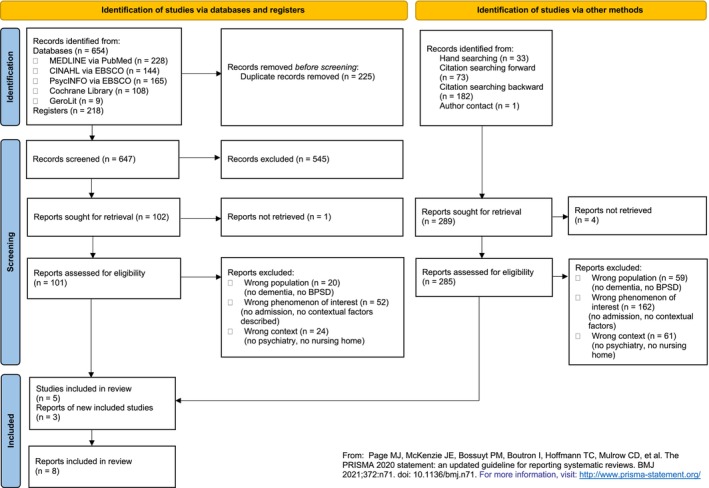
PRISMA 2020 flow diagram of literature searching and screening which included databases, registers and other sources.

Figure 1 shows the PRISMA flow diagram (Page et al. 2021) which visualises the systematic literature search. A total of 872 articles were identified via databases and registers (databases = 654, registers = 218). After removing the duplicates (= 225), 647 articles were screened by title and abstract. A total of 101 potentially eligible papers were subjected to full‐text screening, 5 of which were included. A total of 289 papers were identified via additional research methods (hand search, citation search). Four could not be obtained due to lack of full‐text access or unavailable unpublished papers. A total of 285 potentially eligible papers were screened in full text, and 3 articles could be included so that a total of 8 articles could be integrated and retained for data extraction.

### 5.2 Study Characteristics

A total of 8 studies/articles met the inclusion criteria and are summarised in Table 1.

**TABLE 1:** Characteristics of included studies/articles.

Study characteristics	Sample and setting/context characteristics	Phenomena of interest	Key themes/issues identified for admission context factors
Backhouse et al. 2018 Study design: systematic Review	Participants: Patients with different forms of dementia and different severities of dementia Behaviour that challenges reported by Assessments: NPI (28%, 5/18 studies); CMAI (11%, 2/18 studies); Brief Psychiatric Rating Scale (BPRS) (6%, 1/18 studies); Dementia Behaviour Disturbance Scale (DBDS) (6%, 1/18); 56% (10/18) reported behaviour that challenges without using measures/scales Average age: 78 years Setting/Context: studies from Europe, North America, South‐East Asia and Australia. 67% of the studies reported participants resided in long‐term‐care (12/18 studies reported); 50% of the studies reported participants admitted to psychiatric units (9/18 studies reported)	Factors associated with behavioural crises in dementia Type of behaviours when behavioural crises occur in dementia Coping and resolution strategies of behavioural crises	Aggression/agitation identified as a key behaviour at the point of crisis No long‐term resolution of crises, only short‐term resolution of crises (reported readmissions) Poor evidence on the management of behavioural crises in dementia Crisis resolution interventions are too nonspecific Insufficient information on variables that relate to the facility, care or people with dementia and behavioural crises No reliable data on the management of agitation/aggression in dementia‐related behavioural crises Aggression/agitation are described as crisis triggering key behaviours, but there exist different approaches to manage it Clear, consistent international guidelines with reference to a multicomponent approach (pharmacological and nonpharmacological interventions) could be helpful for crisis management
Pöschel and Spannhorst 2018a Study design: mixed‐method study (document analysis, expert interviews, expert survey, group discussion)	Participants: document analysis:100 patient records, people with dementia (dementia diagnosis reported: 66% Alzheimer's disease, 13% vascular, 1% frontotemporal, 3% Parkinson's disease, 16% other/mixed, 1% not specified); Expert interviews: 2 GPs, 2 care providers from outpatient care services and 2 professional carers; Expert survey and group discussion: 48 general practitioners) Behaviour that challenges: 69% people with dementia and behavioural disturbances at time of admission *n* = 69/100 (document analysis) Average age 80.98 years (document analysis) Setting/Context: Study setting: gerontopsychiatric department (document analysis) in Germany; Outpatient‐inpatient interface (expert interviews); Bielefeld GP Day 2017 (expert survey and group discussion)/housing situation before admission to gerontological psychiatry (document analysis): 32 patients (*n* = 32) came from a professional care setting (31.3% from residential care community (10/100); 68.7% from an inpatient residential and care centre (22/100).	Characterisation of the social, nursing and medical needs of people with dementia at the outpatient‐inpatient interface. This resulted in the dynamic concept of Dementia Care Sensitive Demands (DCSD).	Presence of multiple admission diagnoses/reasons Clarification of the need for nursing support and the clarification of the housing perspective are treatment tasks Patients who have already been treated in gerontological psychiatry indicate an intensive need for treatment or repeatedly decompensated (…) situations. Behavioural problems are considered a reason for decompensation” The understanding of behaviour in need of treatment has changed, which also changes the tasks of psychiatry UV Unsuccessful communication is a central inhibiting factor for cooperation. Lack of knowledge of each other's working methods Unrealistic expectations of patients/relatives towards the professionals or between the professionals themselves Side effects in treatment due to lack of knowledge of each other Active use of legitimising diagnoses to initiate clinical treatment Setting of origin influences the reason for admission/discharge Lack of required care structures, such as specialised care facilities, resulting in waiting times or interregional moves Inability to care for special patients despite agreements and preparation ➔ Revolving door effect Complex needs are based on the presence of dementia (dementia itself + severity of dementia + social environment = time of decompensation or admission) Personality of the actors has an influence on the care of people with dementia Decompensation of the support system leads to admission Gaps in formal communication lead to breaks in care, as lead times are not sufficient for the professional actors
			From the external perspective of the “professional”, the actual need for support cannot be well assessed Acceptance of living and care situations until they decompensate and collapse Admission to geriatric psychiatry is actively used to switch to a different care concept If the cause of psychiatric changes is not recognised and cannot be investigated a referral is made for further clarification Legitimation of admissions by medical reasons, if increased support or assistance is required GP feel helpless in the relationship with their patients with dementia Lack of checklists identifying behaviour that challenges based illnesses that could be treated in advance and reduce inpatient admissions Planned admissions of subacute cases of illness not possible due to a shortage of beds ➔ Shift in treatment towards acute complex cases The resilience of the environment is subject to considerable variation A number of behaviour that challenges remained, despite improvement of symptoms Somatic illnesses provide a treatment‐guiding or treatment‐accompanying factor. Decisions for inpatient admission depend significantly on the care setting and not solely on the presence of the disorder
Pöschel and Spannhorst 2018b Study design: secondary data analysis	Participants: Patients with dementia (100 patient records were reviewed, exclusively from patients with a dementia diagnosis, not specified) Behaviour that challenges: Type and frequency of behaviour that challenges was surveyed at three different times (before admission, at the time of admission, at the time of discharge) Age: Age not specified, but people with dementia were admitted to geriatric psychiatry. It can therefore be assumed that these are people > 60. Setting/Context: Gerontopsychiatric department in Germany; 32 patients (*n* = 32) came from a professional care setting	Differentiation of care‐sensitive need situations Presentation of the complex interplay of social, nursing and medical care needs regarding admission, treatment and perspective development in a gerontological psychiatry department.	The DCSD needs situations include social needs (informal support system, multiprofessional cooperation, psychosocial factors, formal support system); medical needs (somatic diagnoses, psychiatric diagnoses); care needs (mobility, cognition and communication, behaviour and psyche, self‐care, special burdens, everyday life and social contacts, activities outside the home, household management). A decompensation (crisis) in the need situation which cannot be compensated leads to admission to gerontological psychiatry The need situations influence each other and show themselves with different manifestations on the surface or in the background. Admission to psychiatry depends on when and to what extent the care system decompensates. This depends on the stress experience of the different actors, which is also linked to psychosocial factors. The individual perception of stress is linked to psychosocial factors that are shaped by personal relationships, role perceptions and biographical experiences. Psychosocial factors influence behaviour, symptom severity, willingness to provide care and the experience of stress (people with dementia and people around them).
Pöschel et al. 2018 Study design: mixed method study (retrospective explorative document analysis: qualitative analysis using an analysis grid; quantitative analysis by determining the frequencies of reasons for admission/BPSD)	Participants: People with dementia (40 cases from a random sample of 160 treatment cases) who have been admitted to geriatric psychiatry due to behaviour that challenges. Behaviour that challenges: The course of various behaviours was reported. Age: Average age 81.78 years Setting/context: Gerontopsychiatric department with three wards in Germany/30% (*n* = 12) of the patients came from an inpatient care facility.	Presentation of the medical, nursing and social situation before admission, at the time of admission and at discharge, explanation of the differences	People with dementia are discharged with behaviour that challenges although still acute despite intensive inpatient treatment. The number of behavioural problems increased significantly at the time of admission. This led to changes in the level of care required and ultimately to admission or, in persistent cases, to a change in the centre of life. Study shows complex needs of people with dementia at the outpatient‐inpatient interface High chronic and acute somatic disease burden + unstable support system + pronounced need for nursing assistance lead to considerable stress at the nursing level, which significantly influences the decision to admit the patient to psychiatry Referral for inpatient treatment was justified by medical diagnosis: legitimate diagnosis can hide social conflicts Danger to self or others can legitimise admission as accommodation according to BGB (German Civil Code) or PsychKG (Mental Health Act). This is always justified by social and nursing needs in addition to medical needs. Behaviour that challenges was reported as the culmination of multidimensional problems Successful treatment of delirium, but not successful treatment of behaviour that challenges (no complete remission of these behaviours in many cases)
Richler et al. 2023 Study design: unsystematic review, case analysis	Participants: One fictive case (and literature review) with advanced Alzheimer's dementia Behaviour that challenges: Mr. L. had different behaviours that challenges (wandering, intrusive behaviours, paranoia, impulsivity, physical aggression) Age: Mr. L. was 83 years old Setting/context: Mr. L. was admitted from a nursing home to a geriatric psychiatric ward (Country not reported, authors' correspondence address is in Worcester, Massachusetts, USA)	Presentation of the problem that, despite the introduction of Advance Care Planning (ACP), there is a lack of plans for dealing with behaviour that challenges. The problem is described based on the results of a literature analysis and based on the case.	The lack of a management plans for behaviour that challenges lead to stress for the patient, family and treatment team There is uncertainty among care providers (nursing homes) in talking about behaviour that challenges due to lack of knowledge and experience. There is a lack of awareness or knowledge that dementia is progressive and leads to worsening of symptoms, including behaviour that challenges, in the overall course. There is a lack of knowledge that behaviour that challenges is very common in people with dementia and is due to the dementia disease and not to the personality of the patient. There is no cure and no FDA‐approved treatment for behaviour that challenges—many families do not know this and therefore have heightened expectations about the success of the treatment process in relation to behaviour that challenges. There is also a lack of awareness about the complexity of medical decisions that need to be made regarding treatment of behaviour that challenges (including antipsychotic use and mortality risk). Only a limited number of facilities that can handle behaviour that challenges exist. Since COVID‐19 these facilities have become even rarer. Management of behaviour that challenges in long‐term care facilities is complicated by the need to balance sedation and agitation. Discussions about behaviour that challenges (with relatives) only take place when the patient is admitted to a gerontopsychiatric ward for acute stabilisation
Spannhorst et al. 2020 Study design: practice report	Participants: People with dementia Behaviour that challenges: Behaviour that challenges, delirium, depressive or acute psychotic illnesses were reported Age: > 65 Setting/context: Gerontopsychiatry/home or nursing homes in Germany	Presentation of the implementation of inpatient equivalent care at a gerontological psychiatric unit	The clinic setting represents an artificially shielded environment (stabilisation steps achieved there cannot be transferred to the patient's home) Due to the shortage of nursing staff in care homes, it is not possible to treat patients in accordance with the guidelines (S3 guideline on dementia). Aggressive behaviour by others in care homes often leads to inpatient admission as spatial separation is desired. The contact restrictions during the pandemic (ban on visits/ban on staff) led to more admission pressure in gerontological psychiatry.
van Voorden et al. 2024 Study design: mixed method study	Participants/Sample: 13 Units with temporarily admitted Patients with dementia (not specified) and severe behaviour that challenges (16 units were identified, 13 participated) Behaviour that challenges: the participating units included people with dementia and severe behaviour that challenges (not specified) Mean age of the people with dementia in the highly specialised units: from 65 to 82 years Setting/context: Highly specialised units/admitted from regular Dementia Special Care Units (DSCUs), home or mental health care in the Netherlands	Presentation of the general characteristics of highly specialist units (admission/discharge, staffing, physical environment, management of severe behaviour that challenges)	At the time of admission, most patients were taking many different types of psychotropic drugs, often for no good reason In a few cases, the behaviour for which the patients were admitted, classified as serious and challenging, was no longer present after admission. The team's attitude towards the behaviour that challenges seem to play a fundamental role in dealing with people with dementia. Prior to admission, the measures that had been taken thus far were reviewed and advice was often given to prevent admission. Even in highly specialised units, treatment is based on expert opinions rather than guidelines, as these are often not helpful in interpreting behaviour and providing appropriate treatment. Discharge from highly specialised units was difficult in some cases due to a lack of knowledge about what is manageable in DCSUs.
Wetterling 2015 Study design: prospective cross‐sectional study	Participants: 284 (22.4%) out of 1266 cases with insufficient food intake; 214 (75.4%) persons were affected by dementia or delirium in dementia, type of dementia not specified Behaviour that challenges: Insufficient intake of food and liquids was assessed as behaviour that challenges due to the rejection of food and liquids by people with dementia Age > 65 years Setting/context: Clinic for Psychiatry, Psychotherapy and Psychosomatics in Germany/450/1266 (35.5%) from nursing homes	Characterisation of elderly psychiatric patients with food intake disorders	Insufficient food intake was a reason for admission (14.5% of the total sample—not only from homes, not only dementia) Food intake is environment‐related People with dementia have difficulties adapting to nursing home routines Too little attention to the people with dementia, due to lack of time resources Concepts for dealing with refusal to eat exist, but are not sufficient Elderly patients who were admitted from a home were significantly more likely to have insufficient food intake (*p* < 0.001)

This review draws on diverse data sources on contextual factors associated with (re)admission. Study designs varied, and some lacked sufficient details for clear classification. There were three mixed methods studies (Pöschel and Spannhorst 2018a; Pöschel et al. 2018; van Voorden et al. 2024), one systematic review (Backhouse et al. 2018), one secondary data analysis (Pöschel and Spannhorst 2018b), one prospective cross‐sectional study (Wetterling 2015), one unsystematic review combined with a case analysis (Richler et al. 2023), and one practice report (Spannhorst et al. 2020). The types of publications ranged from original research articles and review articles to scientific monographs and opinion papers.

Due to the heterogeneity of the study designs, we concentrated on the study characteristics relevant to our research question: people with dementia; behaviour, that challenges; admission to a psychiatric ward; from a nursing home; and contextual, determining factors. People with dementia were reported differently in the studies/articles. People with varying dementia types and severities, often unspecified (Backhouse et al. 2018; Richler et al. 2023; van Voorden et al. 2024; Wetterling 2015) were included. In some studies/articles, the specific forms of dementia were named, for example, Alzheimer's disease, vascular, frontotemporal, Parkinson's disease, other/mixed (Pöschel and Spannhorst 2018a; Pöschel et al. 2018), or it was a specific case (advanced Alzheimer's dementia) that was focused on (Richler et al. 2023). Some studies/articles have reported complications of dementia, such as delirium (Spannhorst et al. 2020; Wetterling 2015), or psychiatric comorbidities, such as depression or acute psychotic illnesses (Spannhorst et al. 2020; Wetterling 2015). In some studies/articles, the type of behaviour that challenges was not specified in detail (Spannhorst et al. 2020; van Voorden et al. 2024); in some cases, particular behaviours were focused on, for example, the rejection of food and fluids (Wetterling 2015); individual behaviours, such as wandering, intrusive behaviour, impulsivity and physical aggression (Richler et al. 2023); or the occurrence of several behaviours, for example, self‐harming/aggressive behaviour, verbal abnormalities, defensive behaviour, delusions, agitation and others (Backhouse et al. 2018; Pöschel and Spannhorst 2018a, 2018b; Pöschel et al. 2018). Almost all studies/articles reported on admission to a gerontopsychiatric ward (Backhouse et al. 2018; Pöschel and Spannhorst 2018a, 2018b; Pöschel et al. 2018; Richler et al. 2023; Spannhorst et al. 2020; Wetterling 2015): one study detailed a specialised ward for behavioural treatment (van Voorden et al. 2024). Almost all studies/articles reported on admissions to psychiatry from a long‐term inpatient care facility or assisted living community (Backhouse et al. 2018; Pöschel and Spannhorst 2018a, 2018b; Pöschel et al. 2018; Richler et al. 2023; Spannhorst et al. 2020; Wetterling 2015), except for one study that reported on a Dementia Special Care Unit (DSCU), equivalent to a dementia‐specialising nursing home (van Voorden et al. 2024). Five studies reported on the setting in Germany (Pöschel and Spannhorst 2018a, 2018b; Pöschel et al. 2018; Spannhorst et al. 2020; Wetterling 2015), while one systematic review covered Europe, North America, South‐East Asia and Australia (Backhouse et al. 2018). One study originated from the Netherlands (van Voorden et al. 2024), while another did not specify a country; based on the correspondence address in Massachusetts (USA), the results of the case analysis presented suggest a US setting (Richler et al. 2023).

### 5.3 Evidence Level and Quality Appraisal Outcomes

Where possible, the studies/articles were categorised according to the study design categories of the Mixed Methods Appraisal Tool (MMAT) (Hong et al. 2018) though clarity was partly limited by missing information in the articles. In case of reviewer disagreement on study design, a third researcher, an expert in nursing science, was consulted. Four studies were appraised via MMAT: three as mixed methods, one as quantitative description. Owing to a lack of information, three studies/articles were categorised as expert opinions and assessed via the ‘JBI‐Checklist for Textual Evidence: Expert Opinion’ (McArthur et al. 2025), an unsystematic review with case analysis (Richler et al. 2023), a professional article (Pöschel and Spannhorst 2018b) and a practice report (Spannhorst et al. 2020). One systematic review was appraised with the ‘JBI‐Checklist for Systematic Reviews and Research Syntheses’ (Aromataris et al. 2015). The detailed critical appraisal can be found in Tables 2, 3, 4. Table 2 shows only the critical appraisal questions and results of the study designs ‘qualitative’, ‘quantitative descriptive’ and ‘mixed methods’, as only these were relevant. Based on the answers ‘yes’ given in the quality assessment, a score was calculated and documented after each statement (see Supporting Information, Tables S3–S5). The strength of evidence of the studies was classified according to the JBI hierarchies (Joanna Briggs Institute 2013, 2014). The systematic review (Backhouse et al. 2018) was rated level 1 according to the ‘Levels of Evidence for Effectiveness’. Document analyses and the expert survey (Pöschel and Spannhorst 2018a; Pöschel et al. 2018) were assigned level 4 (observational, descriptive studies), as there is no separate level for document analyses. The cross‐sectional study (Wetterling 2015) was classified as level 3 (observational, analytical studies) because groups were compared. Qualitative interviews (Pöschel and Spannhorst 2018a; van Voorden et al. 2024) were rated as level 3 according to the ‘Levels of Evidence for Meaningfulness’. Studies with insufficient methodological reporting were rated as level 5 expert opinions (Pöschel and Spannhorst 2018b; Richler et al. 2023; Spannhorst et al. 2020).

**TABLE 2:** Critical appraisal results for the included studies via the Mixed Method Appraisal Tool (MMAT) (Hong et al. 2018).

Category of study design	Qualitative					Quantitative descriptive					Mixed methods				
Studies	1. Is the qualitative approach appropriate to answer the research question?	2. Are the qualitative data collection methods adequate to address the research question?	3. Are the findings adequately derived from the data?	4. Is the interpretation of results sufficiently substantiated by data?	5. Is there coherence between qualitative data sources, collection, analysis and interpretation?	1. Is the sampling strategy relevant to address the research question?	2. Is the sample representative of the target population?	3. Are the measurements appropriate?	4. Is the risk of nonresponse bias low?	5. Is the statistical analysis appropriate to answer the research question?	1. Is there an adequate rationale for using a mixed methods design to address the research question?	2. Are the different components of the study effectively integrated to answer the research question?	3. Are the outputs of the integration of qualitative and quantitative components adequately interpreted?	4. Are divergences and inconsistencies between quantitative and qualitative results adequately addressed?	5. Do the different components of the study adhere to the quality criteria of each tradition of the methods involved?
Pöschel and Spannhorst 2018a											Y	Y	Y	Y	N
Document analysis						Y	N	Y	C	Y					
Expert interviews	Y	Y	Y	Y	Y										
Expert survey						Y	N	Y	C	C					
Group discussion	Y	Y	C	Y	C										
Pöschel et al. 2018											Y	Y	Y	Y	N
Analysis grid (qualitative)	Y	Y	Y	N	Y										
Frequencies (reasons for admission/BPSD) (quantitative)						Y	N	Y	C	C					
van Voorden et al. 2024											Y	Y	Y	Y	Y
Digital questionnaire						Y	Y	Y	Y	Y					
Interviews	Y	Y	Y	Y	Y										
Environment observation						Y	Y	Y	Y	Y					
Wetterling 2015						Y	N	Y	C	Y					

Abbreviations: C, can't tell; N, no; Y, yes.

^a^ Screening questions not included in the table above.

**TABLE 3:** Critical appraisal results for included studies using the JBI Checklist for Systematic Reviews and Research Synthesis (Aromataris et al. 2015).

Study	Q1 Is the review question clearly and explicitly stated?	Q2 Were the inclusion criteria appropriate for the review question?	Q3 Was the search strategy appropriate?	Q4 Were the sources and resources used to search for studies adequate?	Q5 Were the criteria for appraising studies appropriate?	Q6 Was critical appraisal conducted by two or more reviewers independently?	Q7 Were there methods to minimise errors in data extraction?	Q8 Were the methods used to combine studies appropriate?	Q9 Was the likelihood of publication bias assessed?	Q10 Were recommendations for policy and/or practice supported by the reported data?	Q11 Were the specific directives for new research appropriate?
Backhouse et al. 2018	Y	Y	Y	Y	Y	Y	Y	Y	Y	Y	Y

Abbreviations: N, no; N/A, not applicable; U, unclear; Y, yes.

**TABLE 4:** Critical appraisal results for included studies using the JBI Checklist for textual evidence: expert opinion (McArthur et al. 2025).

Studies	Q1 Is the source of the opinion clearly identified?	Q2 Does the source of opinion have standing in the field of expertise?	Q3 Are the interests of the relevant population the central focus of the opinion?	Q4 Does the opinion demonstrate a logically defended argument to support the conclusions drawn?	Q5 Is there reference to the extant literature?	Q6 Is any incongruence with the literature/sources logically defended?
Pöschel and Spannhorst 2018b	Y	Y	Y	Y	Y	Y
Richler et al. 2023	Y	Y	Y	Y	Y	Y
Spannhorst et al. 2020	Y	Y	Y	Y	Y	Y

Abbreviations: N, no; N/A, not applicable; U, unclear; Y, yes.

### 5.4 Theme: Crisis‐Determining Factors

The most important finding is that crisis situations are the trigger for admission to geriatric psychiatry. The contextual factors associated with (re)admission to geriatric psychiatry are the factors that determine crises. The crisis‐determining factors as the main theme are presented in Table S6 in the Supporting Information, with sub‐themes, categories, key quotes and sources.

The crisis‐determining factors and their backgrounds:

#### 5.4.1 Sub‐Theme: Multifactoriality and ‐Dimensionality of the Dementia and Behaviour That Challenges

This sub‐theme is characterised by the increase of care requirements due to behaviour that challenges, the complex causes behind the behaviour, which require complex treatment, and the limited effective treatment options available.

##### 5.4.1.1 Category: Higher Care Requirements and Decompensating Situations

Pöschel et al. (2018) developed Dementia Care Sensitive Demands (DCSD) in the German context, which describes the interplay of social, medical, and nursing reasons for admitting people with dementia and highlights the complexity of their overall situation.

The complexity of DCSD arises from the interaction between social, medical and nursing care areas; any change requires immediate adjustment, otherwise imbalances and crises can arise (Pöschel and Spannhorst 2018a, 2018b). Behaviour that challenges varies in form and progression, strongly impacts care and leads to decompensation, regardless of the environment (Pöschel and Spannhorst 2018a). In some cases, the number of behavioural problems had increased significantly at the time of admission. This leads to changes in care needs, which results in admission to a psychiatric ward and, in extreme cases, a change in the centre of life. Behaviour that challenges, especially delusions, fears, defensiveness and restlessness, stresses nursing staff and drives admission decisions (Pöschel et al. 2018). Spannhorst et al. (2020) confirm the burden for nursing staff due to the increase in behaviour that challenges, especially aggressive behaviour towards others. Often, outpatient help is not utilised because there is a desire for physical separation from people with dementia. Backhouse et al. (2018) identified certain behaviour that challenges, especially agitation and aggression as main triggers, and delusions, wandering, and hallucinations as common triggers that lead to a crisis for the European, North American, South‐East Asian, and Australian contexts. Pöschel and Spannhorst (2018a, 2018b) confirm this and add that undressing, smearing faeces on walls and reversing the day‐night rhythm lead to decompensation in professional care facilities. They conclude that a demonstrable significant increase in behaviour that challenges is the most common reason for decompensation and justifies admission to geriatric psychiatry.

Readmissions of previously treated patients indicate particularly high treatment needs or repeated decompensation in outpatient care (Pöschel and Spannhorst 2018a).

##### 5.4.1.2 Category: Complex Causes and Complex Treatment Orders

The occurrence of chronic and acute somatic illnesses, an unstable support system and increased care needs in combination appear to lead to behaviour that challenges in DCSD as the tip of multidimensional problems in the German context (Pöschel et al. 2018). Pöschel and Spannhorst (2018a) showed that several patients had one or more acute somatic illnesses at the time of admission, with urinary tract infections being the most common, followed by acute respiratory infections, blood sugar imbalances and cardiovascular problems. In addition to somatic comorbidities, psychiatric comorbidities were also present in people with dementia upon admission. Delirium occurred in a quarter of cases; psychotic symptoms, delusions, or misidentification in a few; depressive syndromes in very few and in a small proportion of cases, the admission diagnosis was unclear. Almost half of the patients had at least two admission diagnoses, a small proportion had three, and social conflicts occurred in very few cases.

Pöschel et al. (2018) critically note that social reasons such as conflict situations were only cited as an explicit reason for admission in a minimal proportion of cases, while aggression towards objects or persons was mentioned significantly more frequently.

Complex causes of behaviour that challenges require complex treatment.

Pöschel and Spannhorst (2018a) mention somatic disorders as accompanying symptoms or guiding factors in geriatric psychiatric treatment. They found that medication adjustments, delirium treatment, and sedation were the most common treatment requests made to geriatric psychiatry. Many cases required dementia diagnosis or clarification of future care and living. Half had at least one additional treatment order; some had several or unclear orders, illustrating the complexity of dementia care needs.

van Voorden et al. (2024) emphasise for the Dutch context that the social and physical environment, in particular, influences the occurrence of behaviour that challenges and that, in some cases, behaviour classified as challenging that leads to admission no longer occurs after admission. Wetterling (2015) suspects that the environment has an influence on behaviour, as insufficient food intake was only observed during inpatient treatment in his findings.

##### 5.4.1.3 Category: Limited Effective Treatment Options

The complexity of behaviour that challenges makes it difficult to provide adequate treatment. Backhouse et al. (2018) mention for the European, North American, South‐East Asian and Australian contexts that there is little high‐quality evidence for the adequate management of behaviour that challenges, there is no clear definition of the interventions used, and there are no indications of long‐term solutions. They conclude this from their systematic review results, in which one third of the studies reported readmissions of patients during the study period.

Pöschel et al. (2018) also showed for the German context that, despite the highly specialised nature of geriatric psychiatry, delirium could be successfully treated, but behaviour that challenges could not. In almost half of the cases, behaviour that challenges was still acute at discharge following intensive inpatient treatment. Pöschel and Spannhorst (2018b) confirm that a significant proportion of behaviour that challenges persists even after an acute phase of illness.

Gerontopsychiatric treatment showed a tendency towards a drastic improvement in symptoms at the end of hospitalisation in most patients, but the variety of behaviour that challenges persisted (Pöschel and Spannhorst 2018a). The limited treatment options for certain challenging behaviours and their consequences are evident in the German cross‐sectional study by Wetterling (2015), which found that the clinical condition of people with dementia who had insufficient food intake was significantly worse.

Richler et al. (2023) report, presumably in the US context, a lack of interventions, FDA‐approved medications and appropriate plans for dealing with behaviour that challenges, which places an emotional burden on those affected, their relatives and caregivers. Relatives are often unaware that behaviour that challenges is part of dementia, that treatments are complex and that psychotropic drugs can increase the risk of death.

Other studies/articles also highlight the complexity of behaviour that challenges and the limited treatment options available.

General practitioners (GPs) in Germany sometimes feel helpless in their relationships with patients with dementia (Pöschel and Spannhorst 2018a). The limited treatment options for behaviour that challenges are also illustrated by the fact that psychotropic drugs are used despite their immense side effects.

Van Voorden et al. (2024) report from their Dutch interview results that many people with dementia take multiple, often unnecessary, psychotropic drugs upon admission. Interviewees wanted to discontinue these drugs but often considered it impossible. Satisfaction was achieved when the dosage was reduced and the reasons for their use were better explained. Treatment was mostly based on expert opinion rather than guidelines, as these were considered unhelpful.

Since there are only limited evidence‐based treatment options available for specific behaviour that challenges, it is necessary to try out different approaches and adapt them to individual needs.

Backhouse et al. (2018) show for the European, North American, South‐East Asian, and Australian contexts that half of the studies reported the use of pharmacological and non‐pharmacological interventions to treat behavioural problems, with almost a third reporting exclusively pharmacological treatment. Spannhorst et al. (2020) highlight the limited effectiveness of interventions in the German context due to the lack of direct transferability of stabilisation measures from geriatric psychiatry to the home environment.

#### 5.4.2 Sub‐Theme: Structural and Systemic Barriers

This sub‐theme highlights the lack of resources at the interface between geriatric psychiatry and nursing homes, which affects staff, time, technical aids, and treatment and nursing home places. It also addresses the strain limits of those involved in the care system, which are intensified by various factors.

##### 5.4.2.1 Category: Care Deficit Due to Lack of Resources

In his German cross‐sectional study, Wetterling (2015) shows that inadequate food intake was significantly more common among people with dementia in nursing homes and led to hospitalisation. He concludes that care deficits in nursing homes are characterised by less attention being paid to people with dementia due to staff shortages and time pressure, which makes it difficult for residents to adapt to the routines there. Spannhorst et al. (2020) mention in their German practice report that a guideline‐oriented (e.g., S3 guideline ‘Dementia’ (DGPPN and DGN 2026)) approach to behaviour that challenges is not possible due to limited staff and time.

According to expert interviews, supply bottlenecks can also be caused by a shortage of beds and multiple transfers in geriatric psychiatry, resulting in long waiting times for admission, which must be bridged. In the group discussion, it became clear that the shortage of beds makes the transfer of subacute cases almost impossible, except in acute cases (Pöschel and Spannhorst 2018a).

Furthermore, there is a shortage of suitable nursing homes for people with severe dementia in Germany. Even facilities with high‐quality care concepts often refuse admission, resulting in long waiting times or a supraregional search for a nursing home. This has an impact on discharge from geriatric psychiatric wards (Pöschel and Spannhorst 2018a). Richler et al. (2023) confirm the difficulty of placing people with dementia and behaviour that challenges in suitable care facilities in the US context. Nursing homes that can adequately deal with such behaviour are in short supply. Since the COVID‐19 pandemic, resources have become even scarcer. The treatment of behaviour that challenges is complicated by the need to find a balance between agitation and sedation.

The care deficits are also evident in the fact that GPs find it difficult to identify symptoms that are masked by behavioural disorders/delirium, as certain technical aids are only available in hospitals (e.g., X‐ray examinations or intravenous antibiotic therapy), which necessitates hospitalisation (Pöschel and Spannhorst 2018a).

##### 5.4.2.2 Category: Capacity Limit of the Care System

Pöschel and Spannhorst (2018a) show that decisions to refer patients to hospital do not depend solely on the presence of an illness, but largely on the current conditions of care. In another article, they point out that the urgency of a referral depends on the capacity of the care facility (Pöschel and Spannhorst 2018b). The expert interviews conducted by Pöschel and Spannhorst (2018a) show that, in addition to the complex needs of people with dementia, the social environment determines ‘when a system “fails”, “no longer works” and referrals become necessary’. The time of admission or discharge depends on the place of origin, for example, residents of nursing homes are admitted later and often discharged earlier, which illustrates the different stress thresholds. The strain is also evident in the fact that people with dementia are transferred to geriatric psychiatric wards in order to prepare for a change in care through physical separation. Despite prior preparations, nursing homes are unable to provide adequate care for certain patients, which can lead to repeated admissions to geriatric psychiatric wards without stable care being achieved. They suspect that the resilience of the respective care contexts differs. What could be compensated for in one environment led to escalations and overload in another.

They also mention in their opinion paper that psychosocial factors, such as relationship and role experiences and one's own biography, can influence behaviour, symptom severity, motivation to provide care, and the actual and perceived burdens of those involved. This applies to people with dementia themselves and to those who care for them (Pöschel and Spannhorst 2018b). van Voorden et al. (2024) show in their Dutch interview results that the stress or tolerance limit of staff towards behaviour that challenges appears to play a fundamental role in dealing with people with dementia. It determines how long and to what extent behaviour that challenges is considered acceptable in a care and support context, whether the behaviour should be stopped immediately or whether the person with dementia should be allowed to express that behaviour freely.

Interviews with German experts make it clear that the stress threshold of the care system has changed, which is reflected in the change in the assessment of the need for admission, that is, behaviour that challenges is tolerated for longer before admission, and the understanding of ‘normal’ behaviour that does not require treatment in psychiatry has also changed, leading to a change in the tasks performed in psychiatry. Another aspect of the strain is becoming apparent through the consideration of social reasons in conjunction with medical and nursing reasons. Socially indicated referrals are legitimised by medical diagnoses in order to obtain clinical treatment. Nursing homes, for example, turn to legal guardians when care is no longer feasible in order to obtain a medically justified referral from a physician. Without a medically justified diagnosis, admission on purely social grounds is not possible (Pöschel and Spannhorst 2018a). Spannhorst et al. (2020) report further challenges that affected patient care in Germany, particularly the implementation of inpatient‐equivalent home treatment (StäB). During the COVID‐19 pandemic, the burden was increased by factors such as visiting bans in nursing homes, which led to higher admission pressure in gerontopsychiatric clinics.

#### 5.4.3 Sub‐Theme: Lack of Effective Interprofessional and Patient‐Centred Collaboration

This sub‐theme illustrates how inadequate communication and differing views, evaluation criteria and practices among professional and non‐professional actors can impair collaboration.

##### 5.4.3.1 Category: Communication Deficits

Interprofessional collaboration is decompensated by a lack of communication, inadequate common goals and the questioning of cooperation (Pöschel and Spannhorst 2018b). In their German expert survey, Pöschel and Spannhorst (2018a) found that poor communication is one of the main reasons for a lack of collaboration. Information not shared requires unnecessary research effort. Communication deficits are characterised by a lack of knowledge about each other's working methods and measures, which can lead to side effects in treatment. Poor formal communication (referral forms, discharge letters, transfer forms) can interrupt care, as the lead times for further care providers are then too short. In their survey of German GPs, they found that there are no checklists for identifying conditions (e.g., infections) that lead to behaviour that challenges. Early detection enables timely outpatient treatment and avoids hospitalisation. When patients are discharged, reliable communication between hospitals and GPs is essential to avoid gaps in care, especially when a new GP is consulted, for example when moving to a nursing home (Pöschel and Spannhorst 2018a). In their review and case analysis, Richler et al. (2023) highlight the inadequate communication about dementia and behaviour‐specific problems in nursing homes for the US context. Discussions about behaviour that challenges, especially about Advance Care Planning (ACP), often take place too late, namely only when people with dementia are admitted to a geriatric psychiatry. In addition, nursing staff avoid such discussions due to a lack of expertise and uncertainty about how to communicate about behaviour that challenges.

Different communication levels can lead to different perceptions, which can promote misunderstandings, both between professional actors and people with dementia when communication skills deteriorate and patient‐centred, adapted communication is lacking (Pöschel and Spannhorst 2018b).

In the results of their interviews with Dutch interviewees, van Voorden et al. (2024) emphasise the importance of cross‐sectoral communication, which should include a critical assessment of the necessity of admission and the measures taken prior to admission, as well as recommendations for avoiding admission.

##### 5.4.3.2 Differences in Views, Evaluation Criteria and Practices Among Stakeholders

In their expert interviews, Pöschel and Spannhorst (2018a) found that interprofessional collaboration breaks down when possible solutions are not accepted, medical professionals do not rely on each other's expert judgement, and too many opinions, discussions and hierarchical behaviour hinder treatment progress. The holistic view of patients can be lost due to the ‘tunnel vision’ of those involved, and the perspective of the ‘expert’ makes it difficult to assess the actual support needs of patients. The standards used by those involved to assess patient risks are difficult to transfer, and the different contexts should not only be considered superficially; the different roles of those involved should also be considered. Another problem in interprofessional collaboration arises when the stakeholders' perception of the problem changes, problems are ignored, or agreements are not adhered to. Different views on treatment and unrealistic expectations between professionals or between patients and their relatives towards professionals make interprofessional and patient‐centred collaboration difficult. Relatives often have different perspectives and evaluation criteria that can influence referral to psychiatry, for example, pathological behaviour of a person with dementia is viewed as purely authoritarian behaviour, or when objectively available psychiatric services are not used out of shame.

Richler et al. (2023) confirm the differing perspectives of professionals and relatives of people with dementia in the US context. They show that relatives often have unrealistic expectations of the treatment process due to a limited understanding of the disease progression and the available treatment options.

The differing views of stakeholders are also evident in the discharge process. In their interviews with Dutch interviewees, van Voorden et al. (2024) found that discharge from specialist clinics appears to be impossible in some cases due to a lack of knowledge about what is possible in regular nursing homes. In some units, nursing staff from the target facility are invited to the clinic to provide mutual support and explain behavioural patterns.

Interprofessional and patient‐centred collaboration can be hindered by the personalities of stakeholders. Different assessment criteria for further care lead, for example, to legal guardians choosing a form of care for people with dementia that corresponds to their own experiences and preferences. In addition, the living and care situations of people with dementia must be accepted until they escalate, despite different perspectives or better assessments by the professionals involved (Pöschel and Spannhorst 2018a).

**FIGURE 2:**
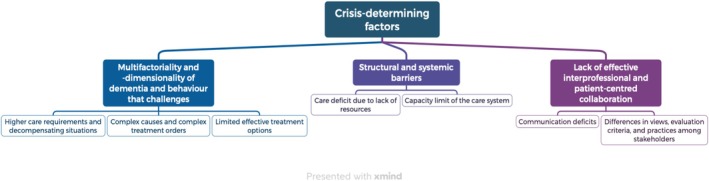
Crisis‐determining factors associated with (re)admission.

The results were generated via the XMind app and are visualised in Figure 2.

## 6 Discussion

A total of eight studies/articles were identified that met the inclusion criteria. It became clear that the contextual factors sought were ‘crisis‐determining factors’. Three sub‐themes were identified: ‘multifactoriality and ‐dimensionality of dementia and behaviour that challenges’, ‘structural and systemic barriers’ and ‘lack of effective interprofessional and patient‐centred collaboration’.

These three sub‐themes highlight the complex causes, effects and limited treatment options for dementia and behaviour that challenges. Structural and systemic barriers significantly limit the care provided to this vulnerable patient group, such as a lack of resources and a system that reaches its limits daily. We identified the lack of effective collaboration between professionals and with relatives as a central and most modifiable issue.

Poor communication seems to be a major reason for the lack of interprofessional collaboration in Germany. The lack of communication means that professional actors are unaware of each other's working methods and measures, which can lead to adverse effects in treatment (Pöschel and Spannhorst 2018a). Romøren et al. (2017) confirm these findings for Norway and report from focus groups with nursing home and hospital doctors that, despite different treatment approaches, there was little communication during transfers and concerns about unnecessary admissions and overtreatment.

These results could indicate that inadequate interprofessional communication has negative effects on the course of patient treatment, including side effects, unnecessary admissions and overtreatment.

van Voorden et al. (2024) emphasise the importance of cross‐sector communication and collaboration for the Netherlands. Prior to admission, a critical assessment of the necessity of admission and the measures already taken is carried out and recommendations are made to avoid admission.

O'Neill et al. (2015) also critically assess the necessity of admission, describe the perceptions of nursing staff in nursing homes and emphasise the importance of the role of nursing staff with regard to admissions and transfers. Nursing staff are confronted with a complex transfer process that requires extensive knowledge and skills, which is why it is necessary to formalise this process and optimise communication and cooperation between those involved.

The results show the importance of early, structured cross‐sector communication. Transfers from nursing homes to hospitals are complex and require specific skills. The decision to transfer patients is particularly difficult in cases of dementia and behaviour that challenges. Early involvement of geriatric psychiatry can provide advisory support and reduce avoidable transfers.

Richler et al. (2023) address the inadequate communication with relatives of people with dementia about behaviour that challenges. Comprehensive, ACP that examines this issue in greater detail takes place far too late, as it is often avoided by nursing staff due to uncertainty about how to deal with behaviour that challenges. They mention different perspectives of professionals and relatives of people with dementia and a limited understanding among relatives of the course of the disease and the available treatment options. The importance of collaboration between nursing staff and relatives is also demonstrated by Havreng‐Théry et al. (2022) who examine the experiences and needs of relatives of nursing home residents in France. Relatives primarily want partnership‐based cooperation and improved communication with nursing home staff, as well as training on their relatives' diseases.

The significance of ACP in nursing homes is also demonstrated by Chambers et al. (2023), who emphasise that integrating ACP into integrated care and quality programmes that specifically support nursing homes has been effective in reducing unplanned admissions, especially in the United Kingdom.

In Germany interprofessional collaboration and treatment progress are significantly disrupted when professional actors do not accept each other's possible solutions or when they do not rely on each other's professional judgements (Pöschel and Spannhorst 2018a). Romøren et al. (2017) complement the negative impact on collaboration with preconceived opinions, negative attitudes and perceptions between Norwegian nursing home and hospital doctors. van Voorden et al. (2024) emphasise that a lack of knowledge about working methods in different institutions can make discharge from specialist clinics in the Netherlands considerably more difficult, which is why they recommend mutual support between institutions. To improve collaboration and understanding of different working methods, common treatment goals are crucial. Müller et al. (2020) evaluated an intervention package to promote interprofessional collaboration between GPs and nursing staff in nursing homes in Germany. They examined factors influencing collaboration and its impact on medical care and avoidable hospital admissions. A key element is the quarterly joint target agreement with documented treatment plans.

To summarise, the results provide important insights into key challenges and possible starting points for future interprofessional and patient‐centred collaboration and the impact on healthcare practice.

### 6.1 Strengths and Limitations

One of the strengths of this integrative review is that the entire search and evaluation process was consistently carried out independently by two people. Excluding the search term ‘nursing home’ increased sensitivity, as we examined more results without explicit reference to nursing homes (title/abstract). Systematic full‐text screening reduced the risk of overlooking relevant studies. We cannot rule out selection bias, as studies with a small sample of nursing home residents or unclear information in the abstract may have gone undetected. Furthermore, we focused on studies in which psychiatric admission actually occurred, which may have excluded potentially relevant studies that only dealt with risk constellations or transitional situations. This restriction was necessary to identify contextual factors directly related to admission, in line with the research objective. In addition, the identified studies often lack important information about the decision‐making process and the organisational and personnel factors in the nursing homes that led to admission. This limits the interpretability of the results and may affect their transferability to other contexts. Moreover, due to insufficient information on the contextual factors of admission, results from studies of lower quality and the authors' interpretations were also considered. Nevertheless, such statements can also provide valuable insights, which scientifically justifies their inclusion in the integrative review. The heterogeneity of the study designs required the use of several instruments for quality assessment. Methodological gaps led in some cases to classification as expert opinion, which limits comparability with other studies. For the sake of transparency, the level of evidence, quality and origin of each statement were indicated (see Supporting Information, Tables S3–S5). The comparison remains methodologically challenging and requires caution in interpretation. Different research approaches and JBI hierarchies make it difficult to compare the studies. As most of them originate from Europe, their international transferability is limited and requires context‐specific evaluation.

## 7 Conclusions and Recommendations

Contextual factors associated with (re)admission to geriatric psychiatry for people with dementia with behaviour that challenges from nursing homes are extremely complex. The development of a crisis in people with dementia and their environment leads to admission to geriatric psychiatry.

This review shows that insufficient effective interprofessional and patient‐centred collaboration is one of the factors determining the crisis. There is a lack of high‐quality studies that explicitly address interprofessional collaboration in behavioural problems associated with dementia at the interface between geriatric psychiatry and nursing homes. Future research should draw on theory‐based approaches such as realistic evaluation to systematically analyse context‐specific determinants, underlying mechanisms and outcomes, and to derive robust programme theories for the prevention of unnecessary psychiatric admissions.

The results highlight contextual factors of admission, mainly from European settings. However, due to contextual differences in healthcare systems and legal frameworks, caution is advised when applying the findings internationally. Future research should specifically examine the extent to which the identified problems and recommendations for action can be transferred to other healthcare systems.

This study shows that inadequate cross‐sector communication can lead to treatment discontinuation, treatment side effects and repeated admissions. To counteract this problem in practice, binding cross‐sector communication and documentation standards should be established, for example, through interoperable digital tools that can make treatment processes more transparent and secure. Future studies should investigate how digital communication tools and structured collaboration models could reduce readmissions and improve the safety of care. Health policy measures should promote binding regional care networks in which nursing homes, GPs and geriatric psychiatric clinics work together in a structured manner, for example, through fixed cooperation agreements, common treatment standards, regular interprofessional case discussions and digital communication channels. The aim is to avoid unnecessary readmissions through coordinated, continuous care.

A lack of effective interprofessional collaboration is evident in the inadequate support provided to nursing staff when dealing with behaviour that challenges. Unclear admission criteria and a lack of knowledge about nursing home capacities lead to avoidable crises and hospitalisations. Specialised ACT (Assertive Community Treatment) teams could provide ongoing training and support for staff. Further studies should investigate how collaboration with mobile psychiatric teams can stabilise the care situation in the present context in order to convince policy makers to provide refinancing.

Behaviour that challenges in nursing homes can trigger psychiatric crises and reduce the quality of life of people with dementia. Early ACP involving relatives is important but requires trained staff and clear standards that include psychiatric emergencies. Future studies should investigate how structured ACP processes influence admission rates and capture the perspectives of all those involved. Policy makers should finance ACP independently of the care setting and enable cross‐sector implementation, including in an international context.

## Author Contributions

**Imane Henni Rached:** conceptualisation (lead); investigation (equal); analysis (equal); visualisation (lead); writing – original draft (lead); writing – review and editing (lead). **Ivonne Ledtermann:** investigation (equal); analysis (equal). **Renate Stemmer:** conceptualisation (supporting); analysis (supporting); writing – review and editing (supporting).

## Funding

This integrative review was supported as part of a larger research project belonging to Imane Henni Rached's doctorate: by the Rheinhessen‐Fachklinik Alzey, where the author works as an APN, and by the Wilhelm Woort‐Stiftung für Alternsforschung as a sponsorship award for a research project on application‐oriented ageing research. There are no conflicts of interest on the part of the supporters.

## Ethics Statement

The authors have nothing to report.

## Consent

The authors have nothing to report.

## Conflicts of Interest

The authors declare no conflicts of interest.

## Supporting information


**Table S1:** Presentation of the search strategies in various databases using the research protocol developed by Hirt and Nordhausen (2022).
**Table S2:** Example search on 03 June 2025 to justify the exclusion of the search component ‘nursing homes’.

**Table S3:** Presentation of quantitative quotations, their transformation, evidence level and quality appraisal score.
**Table S4:** Presentation of qualitative quotations, evidence level and quality appraisal score.
**Table S5:** Presentation of interpretative or opinion‐based quotations, evidence level and quality appraisal score.

**Table S6:** Coding table with the theme, sub‐themes, categories, codes, key quotes and the respective sources.

## Data Availability

The data are contained in the manuscript. The data sets extracted and/or analysed in this study are available on request from the corresponding author.
