# Supplementary material for: From Nursing Homes to Geriatric Psychiatry: Contextual Factors Associated With the Admission of People With Dementia and Behaviour That Challenges—An Integrative Review
Source: Nurs Open. 2026 May 16;13(5):e70592. doi: 10.1002/nop2.70592 (PMC13179823; doi:10.1002/nop2.70592)
Supplement: Supplementary file 1 — Table S1: Presentation of the search strategies in various databases using the research protocol developed by Hirt and Nordhausen (2022). Table S2: Example search on 03 June 2025 to justify the exclusion of the search component ‘nursing homes’. [file NOP2-13-e70592-s001.docx]

Table S1 Presentation of the search strategies in various databases using the research protocol developed by Hirt and Nordhausen (2022)

| **Search component** | **Search string**  **MEDLINE via PubMed** | **Search string**  **CINAHL via EBSCO** | **Search string**  **PsychINFO** | **Search string**  **Cochrane Library** | **Search string**  **GeroLit** |
| --- | --- | --- | --- | --- | --- |
| **Search component 1 Dementia (Population)** | (dement*[Title/Abstract]) OR (Alzheimer*[Title/Abstract])) OR ("Lewy bod*”[Title/Abstract])) OR (Frontotemporal*[Title/Abstract])) or ("pick disease*"[title/abstract])) OR "pick’s disease*"[Title/Abstract] or ("dementia"[MeSH Terms]) | AB dement* OR TI dement* OR TI Alzheimer* OR AB Alzheimer* OR TI "Lewy bod*" OR AB "Lewy bod*" OR TI Frontotemporal* OR AB Frontotemporal* OR TI “pick disease*” OR AB “pick disease*” or TI “pick’s disease*” OR TI “pick’s disease*” OR MH dementia OR MH dementia patients | AB dement* OR TI dement* OR TI Alzheimer* OR AB Alzheimer* OR TI "Lewy bod*" OR AB "Lewy bod*" OR TI "Frontotemporal*" OR AB "Frontotemporal*" OR TI “pick disease*” OR AB “pick disease*” or TI “pick’s disease*” OR TI “pick’s disease*” OR **DE "Dementia"** OR DE "AIDS Dementia Complex" OR DE "Dementia with Lewy Bodies" OR DE "Presenile Dementia" OR DE "Pseudodementia" OR DE "Semantic Dementia" OR DE "Senile Dementia" OR DE "Vascular Dementia" OR DE "Presenile Dementia" OR DE "Alzheimer's Disease" OR DE "Creutzfeldt Jakob Syndrome" OR DE "Picks Disease" | (dement*):ti,ab,kw OR (Alzheimer*):ti,ab,kw OR  ("lewy body"):ti,ab,kw OR OR  ("lewy bodies"):ti,ab,kw OR (frontotemporaL*):ti,ab,kw OR ("Pick disease"):ti,ab,kw  OR ("Pick diseases"):ti,ab,kw  OR ("Pick’s disease"):ti,ab,kw OR  ("Pick’s diseases"):ti,ab,kw | *DementIA [all] OR *Demenz[all] OR Demen*[all] OR alzheimer*[all] or “lewy bod*”[all] OR frontotemporal*[all] or “pick’s disease*”[all] OR “PICK DIsease*” [all] OR  Demenz [SWW] or  Alzheimer Demenz [SWW] or  Altersdemenz[SWW] or  senile demenz[SWW] or  ALzheimer-Krankheit [SWW] |
|  | AND | AND | AND | AND | AND |
| **Search component 2 Behaviour that challenges (Population)** | (behavio*[Title/Abstract]) or (neuropsychiatric*[Title/Abstract]) or BPSD[Title/Abstract] or ("conduct disorder*"[Title/Abstract])) OR (misconduct*[Title/Abstract])) OR ("behavior and behavior mechanisms"[MeSH Terms]) | TI behavio* OR AB behavio* OR ti neuropsychiatric* or Ab neuropsychiatric* or ti bpsd or ab bpsd or TI "Conduct*" OR AB "Conduct*" Or MH behavior and behavior mechanisms OR behavioral symptoms or psychiatric emergencies | TI behavio* OR AB behavio* OR ti neuropsychiatric* or Ab neuropsychiatric* or ti bpsd or ab bpsd or TI Conduct* OR AB Conduct* OR **DE "Emotional States"** OR DE "Affection" OR DE "Agitation" OR DE "Alienation" OR DE "Ambivalence" OR DE "Anger" OR DE "Anxiety" OR DE "Apathy" OR DE "Aversion" OR DE "Belonging" OR DE "Bereavement" OR DE "Boredom" OR DE "Catastrophizing" OR DE "Compassion" OR DE "Contentment" OR DE "Depression (Emotion)" OR DE "Disappointment" OR DE "Disgust" OR DE "Dissatisfaction" OR DE "Distress" OR DE "Doubt" OR DE "Embarrassment" OR DE "Emotional Exhaustion" OR DE "Emotional Trauma" OR DE "Empathy" OR DE "Enthusiasm" OR DE "Euphoria" OR DE "Euthymia" OR DE "Fear" OR DE "Frustration" OR DE "Gratitude" OR DE "Greed" OR DE "Grief" OR DE "Guilt" OR DE "Happiness" OR DE "Helplessness" OR DE "Homesickness" OR DE "Hope" OR DE "Hopelessness" OR DE "Jealousy" OR DE "Loneliness" OR DE "Love" OR DE "Mania" OR DE "Mental Confusion" OR DE "Morale" OR DE "Optimism" OR DE "Passion" OR DE "Pessimism" OR DE "Pleasure" OR DE "Pride" OR DE "Psychological Capital" OR DE "Psychological Engagement" OR DE "Regret" OR DE "Restlessness" OR DE "Sadness" OR DE "Shame" OR DE "Solidarity" OR DE "Suffering" OR DE "Suspicion" OR DE "Sympathy"  OR  **DE "Behavior"** OR DE "Adaptive Behavior" OR DE "Adjunctive Behavior" OR DE "Adolescent Behavior" OR DE "Animal Behavior" OR DE "Antisocial Behavior" OR DE "Approach Avoidance" OR DE "Approach Behavior" OR DE "Attachment Behavior" OR DE "Avoidance" OR DE "Behavior Change" OR DE "Behavior Problems" OR DE "Child Behavior" OR DE "Childhood Play Behavior" OR DE "Choice Behavior" OR DE "Classroom Behavior" OR DE "Compulsions" OR DE "Consequence" OR DE "Consumer Behavior" OR DE "Coping Behavior" OR DE "Coronary Prone Behavior" OR DE "Daily Activities" OR DE "Drinking Behavior" OR DE "Driving Behavior" OR DE "Drug Usage" OR DE "Eating Behavior" OR DE "Endurance" OR DE "Exploratory Behavior" OR DE "Food Preparation" OR DE "Habits" OR DE "Health Behavior" OR DE "Hoarding Behavior" OR DE "Human Nature" OR DE "Illness Behavior" OR DE "Individual Differences" OR DE "Instinctive Behavior" OR DE "Instrumentality" OR DE "Intraindividual Variability" OR DE "Lifestyle" OR DE "Obscenity" OR DE "Performance" OR DE "Planned Behavior" OR DE "Predisposition" OR DE "Pro Environmental Behavior" OR DE "Productivity" OR DE "Psychological Reactance" OR DE "Psychosexual Behavior" OR DE "Reasoned Action" OR DE "Resistance" OR DE "Responses" OR DE "Sedentary Behavior" OR DE "Self-Defeating Behavior" OR DE "Social Behavior" OR DE "Stereotyped Behavior" OR DE "Voting Behavior" OR DE "Wandering Behavior" OR DE "Social Behavior" OR DE "Activism" OR DE "Aggressive Behavior" OR DE "Animal Social Behavior" OR DE "Caring Behaviors" OR DE "Competition" OR DE "Compliance" OR DE "Conformity (Personality)" OR DE "Contagion" OR DE "Criticism" OR DE "Deception" OR DE "Followership" OR DE "Gambling" OR DE "Help Seeking Behavior" OR DE "Interspecies Interaction" OR DE "Involvement" OR DE "Leadership" OR DE "Leadership Style" OR DE "Militancy" OR DE "Nurturance" OR DE "Organizational Behavior" OR DE "Privacy" OR DE "Prosocial Behavior" OR DE "Racial and Ethnic Relations" OR DE "Reciprocity" OR DE "Respect" OR DE "Responsibility" OR DE "Retaliation" OR DE "Risk Taking" OR DE "Rudeness" OR DE "Self-Presentation" OR DE "Social Acceptance" OR DE "Social Adjustment" OR DE "Social Approval" OR DE "Social Change" OR DE "Social Cognition" OR DE "Social Communication" OR DE "Social Connectedness" OR DE "Social Demonstrations" OR DE "Social Dominance" OR DE "Social Drinking" OR DE "Social Exclusion" OR DE "Social Facilitation" OR DE "Social Functioning" OR DE "Social Groups" OR DE "Social Identity" OR DE "Social Inclusion" OR DE "Social Interaction" OR DE "Social Loafing" OR DE "Social Networks" OR DE "Social Perception" OR DE "Social Reinforcement" OR DE "Social Resources" OR DE "Social Responsibility" OR DE "Social Skills" OR DE "Social Withdrawal" OR DE "Behavior Disorders" OR DE "Conduct Disorder" OR DE "Disruptive Behavior Disorders" OR DE "Impulse Control Disorders" OR DE "Kleptomania" OR DE "Oppositional Defiant Disorder" OR DE "Pyromania" OR DE "Self-Destructive Behavior" | (behavio*):ti,ab,kw OR  OR (neuropsychiatric*):ti,ab,kw or (bpsd):ti,ab,kw or (conduct*):ti,ab,kw | Behavio* [all] or conduct* [all] or neuropsychiatr* [all] or bpsd[all] or Verhalten*[all] or *Verhalten[all] or Verhaltensstörung [SWW] or  Verhalten [SWW] or  herausforderndes verhalten [SWW] or  Agitation [SWW] or  depression [SWW] or  altersdepression [SWW] or  Aggression [SWW] or  aggressionen [SWW] or  aggressivität [SWW] or  emotion [SWW] or  gefühl [SWW] or  gewalt [SWW] or  Soziale Norm [SWW] or  Affektive Störung [SWW] or  Delirium [SWW] or  Psychose [SWW] or  Verwirrtheitszustand [SWW] or  Sozialverhalten [SWW] or  schlafstörung [SWW] or  schlafstörungen [SWW] |
|  | AND | AND | AND | AND | AND |
| **Search component 3 (Re)admission**  **(Phenomenon of interest)** | (Admission*[Title/Abstract]) OR (Admitted[Title/Abstract])) OR (readmitted[Title/Abstract])) OR (Readmission*[Title/Abstract])) OR (Referral*[Title/Abstract])) OR ("Patient transfer*"[Title/Abstract])) OR ("CLIENT transfer*"[Title/Abstract]))OR (Hospitali*[Title/Abstract])) OR (Rehospitali*[Title/Abstract])) OR ("referral and consultation"[MeSH Terms])) OR ("hospitalization"[MeSH Terms]) | TI Admission* OR AB Admission* OR TI Admitted OR AB Admitted OR TI readmitted OR AB readmitted OR TI Readmission* OR AB Readmission* OR TI Referral* OR AB Referral* OR TI "Patient transfer*" OR AB "Patient transfer*" OR TI "CLIENT transfer*" OR AB "CLIENT transfer*" OR TI Hospitali* OR AB Hospitali* OR TI Rehospitali* OR AB Rehospitali* OR Mh referral and consultation OR MH hospitalization OR MH hospitalization of older persons OR MH READMISSION | TI Admission* OR AB Admission* OR TI Admitted OR AB Admitted OR TI readmitted OR AB readmitted OR TI Readmission* OR AB Readmission* OR TI Referral* OR AB Referral* OR TI Hospitali* OR AB Hospitali* OR TI Rehospitali* OR AB Rehospitali* OR TI "client transfer*" OR AB "client transfer*" OR TI "patient transfer*" OR AB "patient transfer*" OR  DE "Professional Referral" OR DE "Client Transfer"  OR  **(DE "Hospitalization"** OR DE "Hospital Admission" OR DE "Hospital Discharge" OR DE "Mental Health Commitment" OR DE "Psychiatric Hospitalization" OR DE "Hospital Admission" OR DE "Psychiatric Hospital Admission" OR DE "Hospital Discharge" OR DE "Psychiatric Hospital Discharge" OR DE "Psychiatric Hospitalization" OR DE "Psychiatric Hospital Admission" OR DE "Psychiatric Hospital Discharge" OR DE "Psychiatric Hospital Readmission") OR (DE "Hospitalized Patients") | (Admission*):ti,ab,kw OR (Admitted):ti,ab,kw OR (readmitted):ti,ab,kw OR (Readmission*):ti,ab,kw OR (Referral*):ti,ab,kw OR ("Patient transfer"):ti,ab,kw OR ("Patient transfers"):ti,ab,kw OR (hospitali*):ti,ab,kw OR (REhospitali*):ti,ab,kw | admission*[all] or admitted[all] or readmission*[all] or referral*[all] or “patient transfer*”[all] or “CLIENT transfer*”[ALL] OR patiententransfer*[all] hospitali*[all] or rehospitali*[all] or Einweisung*[all] or *einweisung[all] or eingewiesen*[all] or Weiterleitung*[all] or Überleitung*[all] or *überleitung[all] or  EInweisung [SWW] or  EInweisungen [SWW] or  EInweisungskriterium [SWW] or  Einweisungskriterien [SWW] |
|  | AND | AND | AND | AND | AND |
| **Search component 4 Geriatric psychiatry (Context)** | (psychiatr*[Title/Abstract]) OR (Gerontopsychiatr*[Title/Abstract])) OR (Geropsychiatr*[Title/Abstract])) OR ("Mental hospital*"[Title/Abstract])) OR (Psychogeriatric*[Title/Abstract])) OR ("hospitals, psychiatric"[MeSH Terms])) OR ("emergency services, psychiatric"[MeSH Terms])) OR ("psychiatry"[MeSH Terms]) | TI psychiatr* OR AB psychiatr* OR TI Gerontopsychiatr* OR AB Gerontopsychiatr* OR TI Geropsychiatr* OR AB Geropsychiatr* OR TI "Mental hospital*" OR AB "Mental hospital*" OR TI Psychogeriatric* OR AB Psychogeriatric* OR MH emergency services, psychiatric OR MH psychiatry OR MH psychiatric units OR MH hospitals, psychiatric | TI psychiatr* OR AB psychiatr* OR TI Gerontopsychiatr* OR AB Gerontopsychiatr* OR TI Geropsychiatr* OR AB Geropsychiatr* OR TI "Mental hospital*" OR AB "Mental hospital*" OR TI Psychogeriatric* OR AB Psychogeriatric* OR  (((**DE "Psychiatry"** OR DE "Adolescent Psychiatry" OR DE "Biological Psychiatry" OR DE "Child Psychiatry" OR DE "Community Psychiatry" OR DE "Consultation Liaison Psychiatry" OR DE "Forensic Psychiatry" OR DE "Geriatric Psychiatry" OR DE "Military Psychiatry" OR DE "Neuropsychiatry" OR DE "Orthopsychiatry" OR DE "Social Psychiatry" OR DE "Telepsychiatry" OR DE "Transcultural Psychiatry") OR (**DE "Psychiatric Hospitals"** OR DE "Psychiatric Units")) OR (**DE "Psychiatric Clinics"))** OR (**DE "Emergency Services"** OR DE "Crisis Intervention Services") | (psychiatr*):ti,ab,kw OR (gerontopsychiatr*):ti,ab,kw OR (geropsychiatr*):ti,ab,kw OR ("mental hospital"):ti,ab,kw OR ("mental hospitals"):ti,ab,kw OR (psychogeriatric*):ti,ab,kw | *Psychiatr*[all] or  “mental hospital*”[all] or psychogeriatr*[all] or  gerontopsychiatrie[SWW] or  gerontopsychiatrische versorgung[SWW] or  psychiatrie[SWW] or  alterspsychiatrie[SWW] or  psychiatrische versorgung[SWW] or  psychiatrisches krankenhaus[SWW] or  psychosoziale versorgung[SWW] |

**Reasons for (individual) search techniques:**

In PsychINFO inclusion of subordinate keywords is only possible using ‘Explode’, which is why these are visible in the search string through OR links. In MEDLINE and CINAHL, subordinate keywords are automatically included when the parent keyword is selected, which is why they are not visible in the search string.

Table S2 Example search on 03 June 2025 to justify the exclusion of the search component ‘nursing homes’.

| **Search string including search component „nursing homes“ in MEDLINE** | **Results** | **Reason for excluding** |
| --- | --- | --- |
| (("dement*"[Title/Abstract] OR "alzheimer*"[Title/Abstract] OR "lewy bod*"[Title/Abstract] OR "frontotemporal*"[Title/Abstract] OR "pick disease*"[Title/Abstract] OR "pick s disease*"[Title/Abstract] OR "dementia"[MeSH Terms]) AND ("behavio*"[Title/Abstract] OR "neuropsychiatric*"[Title/Abstract] OR "BPSD"[Title/Abstract] OR "conduct disorder*"[Title/Abstract] OR "misconduct*"[Title/Abstract] OR "behavior and behavior mechanisms"[MeSH Terms]) AND ("admission*"[Title/Abstract] OR "admitted"[Title/Abstract] OR "readmitted"[Title/Abstract] OR "readmission*"[Title/Abstract] OR "referral*"[Title/Abstract] OR "patient transfer*"[Title/Abstract] OR "client transfer*"[Title/Abstract] OR "hospitali*"[Title/Abstract] OR "rehospitali*"[Title/Abstract] OR "referral and consultation"[MeSH Terms] OR "hospitalization"[MeSH Terms]) AND ("psychiatr*"[Title/Abstract] OR "gerontopsychiatr*"[Title/Abstract] OR "geropsychiatr*"[Title/Abstract] OR "mental hospital*"[Title/Abstract] OR "psychogeriatric*"[Title/Abstract] OR "emergency services, psychiatric"[MeSH Terms] OR "psychiatry"[MeSH Terms]) AND ("Long-Term Care"[MeSH Terms] OR "Residential Facilities"[MeSH Terms])) AND ((fha[Filter]) AND (humans[Filter]) AND (english[Filter] OR german[Filter]) AND (middleaged[Filter] OR aged[Filter] OR 80andover[Filter]) AND (2013:2025[pdat])) | **24** | The search component “nursing homes” was excluded because it restricted the search too much and would have significantly reduced the number of relevant hits. Instead, nursing homes were identified in accordance with the sensitive search principle by manually reviewing abstracts and full texts. |
| **Search string excluding search component „nursing homes“ in MEDLINE** | **Results** |  |
| (("dement*"[Title/Abstract] OR "alzheimer*"[Title/Abstract] OR "lewy bod*"[Title/Abstract] OR "frontotemporal*"[Title/Abstract] OR "pick disease*"[Title/Abstract] OR "pick s disease*"[Title/Abstract] OR "dementia"[MeSH Terms]) AND ("behavio*"[Title/Abstract] OR "neuropsychiatric*"[Title/Abstract] OR "BPSD"[Title/Abstract] OR "conduct disorder*"[Title/Abstract] OR "misconduct*"[Title/Abstract] OR "behavior and behavior mechanisms"[MeSH Terms]) AND ("admission*"[Title/Abstract] OR "admitted"[Title/Abstract] OR "readmitted"[Title/Abstract] OR "readmission*"[Title/Abstract] OR "referral*"[Title/Abstract] OR "patient transfer*"[Title/Abstract] OR "client transfer*"[Title/Abstract] OR "hospitali*"[Title/Abstract] OR "rehospitali*"[Title/Abstract] OR "referral and consultation"[MeSH Terms] OR "hospitalization"[MeSH Terms]) AND ("psychiatr*"[Title/Abstract] OR "gerontopsychiatr*"[Title/Abstract] OR "geropsychiatr*"[Title/Abstract] OR "mental hospital*"[Title/Abstract] OR "psychogeriatric*"[Title/Abstract] OR "emergency services, psychiatric"[MeSH Terms] OR "psychiatry"[MeSH Terms])) AND ((fha[Filter]) AND (humans[Filter]) AND (english[Filter] OR german[Filter]) AND (middleaged[Filter] OR aged[Filter] OR 80andover[Filter]) AND (2013:2023[pdat])) | **237** |  |
