# Supplementary material for: From Nursing Homes to Geriatric Psychiatry: Contextual Factors Associated With the Admission of People With Dementia and Behaviour That Challenges—An Integrative Review
Source: Nurs Open. 2026 May 16;13(5):e70592. doi: 10.1002/nop2.70592 (PMC13179823; doi:10.1002/nop2.70592)
Supplement: Supplementary file 2 — Table S3: Presentation of quantitative quotations, their transformation, evidence level and quality appraisal score. Table S4: Presentation of qualitative quotations, evidence level and quality appraisal score. Table S5: Presentation of interpretative or opinion‐based quotations, evidence level and quality appraisal score. [file NOP2-13-e70592-s003.docx]

Table S3 Presentation of quantitative quotations, their transformation, evidence level and quality appraisal score

| **Study/article** | **Quantitative findings (quotations)** | **Transformation in „qualitised“ data** | **Level of evidence/ quality appraisal score** |
| --- | --- | --- | --- |
| Backhouse et al., 2018,  systematic review | Agitation/aggression was the most cited cause of crisis with all 18 (100%) studies (p. 109) | Agitation and aggression were described in all included studies as key triggers for crisis situations**.** | JBI level 1, 100% JBI Checklist for Systematic Reviews and Research Syntheses |
|  | ‘Delusions were cited by 13/18 (72%) of studies, wandering/absconding 12/18 (67%), and hallucinations 11/18 (61%)‘ (p. 107). | Delusions, running away or wandering and hallucinations were described in most of the studies included. | JBI level 1, 100% JBI Checklist for Systematic Reviews and Research Syntheses |
|  | ‘Seven out of the 18 studies (39%) reported patients in the sample being re-admitted to the institutions within the study timeline or who had had previous admissions‘ (p. 107). | Several studies reported that patients had been re-admitted to institutions during the study period or had a history of previous admissions. | JBI level 1, 100% JBI Checklist for Systematic Reviews and Research Syntheses |
|  | ‘Nine out of 18 studies (50%) reported using both pharmacological and non-pharmacological interventions and 5/18 (28%) used pharmacological interventions only‘ (p. 109). | Half of the studies described the use of both pharmacological and non-pharmacological interventions to manage behavioural problems and almost a third used only pharmacological measures. | JBI level 1, 100% JBI Checklist for Systematic Reviews and Research Syntheses |
| **Pöschel & Spannhorst, 2018a,**  quantitative part of mixed-method study (document analysis) | ‘The most common admission diagnosis was challenging behaviour, with 50 cases, followed by delirium and confusion (26 cases) [...], followed by 11 cases with the symptom complex psychotic symptoms, delusions or misidentification of persons.  ‘Depressive syndrome was reported in 4 cases. In 11 cases, the admission diagnosis was unclear after reviewing the documents.‘  ‘It was noteworthy that 47 of the 100 patient cases had at least two of the admission diagnoses mentioned, and in 11 cases, 3 admission diagnoses were even mentioned [...] social conflicts also in only 3 cases‘ (p. 98) | The most common diagnosis on admission was challenging behaviour, accounting for half of cases, followed by delirium and confusion in about a quarter of cases, [...], followed by a smaller proportion of cases with the symptom complex ‘psychotic symptoms, delusions or misidentification of persons’  Depressive syndrome was reported in a very small proportion of cases. In a small proportion of cases, the admission diagnosis was unclear after reviewing the records.  It was noteworthy that almost half of the patient cases had at least two of the admission diagnoses mentioned, and in a small proportion of cases, three admission diagnoses were even mentioned, [...] social conflicts also in a small proportion of cases | JBI level 4, 60% MMAT |
| **Study/article** | **Quantitative findings (quotations)** | **Transformation in „qualitised“ data** | **Level of evidence/ quality appraisal score** |
| **Pöschel & Spannhorst, 2018a,**  quantitative part of mixed-method study (document analysis) | ‘The treatment order evident from the referral documents concerned in the majority of cases the complex of ‘medication adjustment / delirium treatment / sedation’ (74 of the 100 cases).‘  ‘In 41 cases, differential diagnosis of dementia was requested, and in 37 cases, clarification of further support needs and residential prospects was considered a task for inpatient treatment.‘  ‘In 15 cases, the treatment order was unclear according to the available documents.‘  ‘In 50 of the 100 cases, there was at least one second treatment order [...]. In 19 cases, three or more treatment orders were identified‘ (p. 98-99). | In most cases, inpatient admissions were primarily initiated due to the need for medical intervention, such as medication adjustment, delirium management, or sedation.  In addition, referrals often included requests for diagnostic clarification of dementia and for assessing future care and living arrangements. Several cases reflected overlapping treatment priorities.  In a low proportion of the cases, the treatment mandate was unclear according to the available documents.  In half of the cases, there was at least one second treatment order [...]. In a small proportion of cases, three or more treatment orders were identified. | JBI level 4, 60% MMAT |
|  | ‘Of the n=100 cases examined, 38 had one or more acute somatic illnesses at the time of admission‘ (p. 88).  ‘Urinary tract infections were the most common illness, with a total of 17 cases, followed by 10 cases of acute respiratory diseases, 8 acute metabolic diseases (mainly blood sugar imbalances) and 8 acute cardiovascular diseases‘ (p. 88). | A moderate proportion of patients had one or more acute somatic illnesses at the time of admission.  Urinary tract infections were the most common acute illness followed by acute respiratory infections, blood sugar imbalances and cardiovascular problems. | JBI level 4, 60% MMAT |
| **Pöschel & Spannhorst, 2018a,**  quantitative part of mixed-method study (expert survey) | ‘52% of respondents [GPs] agreed fully or partially with the statement that they sometimes feel helpless in their relationship with their patients suffering from dementia‘ (p. 115). | About half of general practitioners agreed that they sometimes feel helpless in their relationship with their patients suffering from dementia. | JBI level 4, 40% MMAT |
| **Study/article** | **Quantitative findings (quotations)** | **Transformation in „qualitised“ data** | **Level of evidence/ quality appraisal score** |
| **Pöschel et al., 2018,**  quantitative part of mixed-method study (document analysis | ‘Despite intensive inpatient treatment, behavioural disorders were still documented as acute in 9 out of 20 cases at the time of discharge‘ (p. 152). | In almost half of the cases, the behavioural disorders were still acute at the time of discharge after intensive inpatient treatment. | JBI level 4, 40% MMAT |
|  | ‘Of particular interest is the progression of nursing care needs in the area of behavioural problems, which led to admission and, in some cases, to a change in the centre of life. [...] Compared to the period before admission (t0), the number of behavioural problems increased tenfold at the time of admission (t1)‘ (p. 153). | The number of behavioural problems increased significantly at the time of admission. This led to changes in the level of care required and ultimately to admission or, in persistent cases, to a change in the centre of life. | JBI level 4, 40% MMAT |
| **Wetterling, 2015,**  prospective cross-sectional study | ‘Inadequate food intake was significantly more common in cases admitted from a nursing home (p < 0.001). Inadequate food intake was also more frequently the reason for admission among nursing home residents (23.8% vs. 9.4%; p < 0.001)‘ (p. 43). | Inadequate food intake was found to be strikingly common among nursing home residents and was significantly more often the reason for hospital admission than among people with dementia living in other types of accommodation. | JBI level 3, 60% MMAT |
|  | ‘At discharge, the clinical condition was significantly worse in the group with inadequate food intake (CGI change: 6 or 7) (6.7% vs. 1.8%; Chi2 18.3; df = 1; p = 0.001)‘ (p. 44). | At discharge, the clinical condition was significantly worse in the group with inadequate food intake. | JBI level 3, 60% MMAT |

Table S4 Presentation of qualitative quotations, evidence level and quality appraisal score

| **Study/article** | **Qualitative findings** | **Level of evidence/ quality appraisal score** |
| --- | --- | --- |
| **Pöschel & Spannhorst, 2018a,** qualitative part of mixed-method study (expert interviews) | ‘Behavioural problems are considered a cause of decompensation, regardless of the structure of the care environment’ (p. 125). | JBI level 3, 100% MMAT |
|  | ‘Psychiatry is changing in its tasks, as the concept of ‘normal’ behaviour, and therefore behaviour that does not require treatment, has expanded‘ (p. 127). | JBI level 3, 100% MMAT |
|  | ‘Experts cite poor communication as a key factor hindering cooperation.  If information is required but not available, an unnecessary amount of resources must be spent on research‘ (p. 132). | JBI level 3, 100% MMAT |
|  | ‘In addition to communication, other factors are also mentioned, such as waiting times for treatment, which need to be bridged‘ (p. 132). | JBI level 3, 100% MMAT |
|  | ‘These include, for example, a lack of knowledge at the interfaces about how the other party works [...] or a disruption in cooperation between actors that cannot be resolved‘ (p. 132). | JBI level 3, 100% MMAT |
|  | ‘Factors contributing to failure include unrealistic expectations of professionals on the part of patients and their relatives, or between professionals themselves‘ (p. 132). | JBI level 3, 100% MMAT |
|  | ‘Further problems can arise from the tunnel vision of the actors themselves, which leads to a loss of holistic perspective‘ (p. 133). | JBI level 3, 100% MMAT |
|  | ‘Side effects may occur during treatment if one practitioner is unaware of the other's actions, as there is no communication between them‘ (p. 133). | JBI level 3, 100% MMAT |
|  | ‘According to experts, admission for purely social reasons is not possible if there is no medically justified diagnosis for admission‘ (p. 147). | JBI level 3, 100% MMAT |
|  | ‘In accordance with the guidelines for access to services, this [a socially indicated referral] is actively used in the context of referrals, through legitimate diagnoses, to initiate services such as clinical treatment‘ (p. 134). | JBI level 3, 100% MMAT |
|  | ‘However, the setting of origin also has an influence on the timing or reason for admission or discharge. Residents of nursing homes are admitted later and often discharged earlier than patients from home settings‘ (p. 134). | JBI level 3, 100% MMAT |
| **Study/article** | **Qualitative findings** | **Level of evidence/ quality appraisal score** |
| **Pöschel & Spannhorst, 2018a,** qualitative part of mixed-method study (expert interviews) | ‘However, the lack of necessary care facilities, such as specialised nursing homes, can also influence discharge, resulting in waiting times or moves to other regions‘ (p. 134). | JBI level 3, 100% MMAT |
|  | ‘Another variant arises when, despite agreements and preparations, facilities are unable to care for special patients, resulting in a revolving door situation‘ (p. 134). | JBI level 3, 100% MMAT |
|  | ‘A breakdown in cooperation between professional actors [...] when possible solutions are not accepted [...]. Another aspect involves a shift in how actors perceive themselves and each other in relation to problems, or when agreements are changed or not adhered to autonomously. This also includes failing to recognise or acknowledge emerging problem situations or seeking and accepting help at a very late stage‘ (p. 135). | JBI level 3, 100% MMAT |
|  | ‘Too many discussions, opinions and hierarchical interprofessional behaviour are hindering progress‘ (p. 136). | JBI level 3, 100% MMAT |
|  | ‘Psychological phenomena include obstructive role behaviour (misinterpreting pathological behaviour in a patient as merely authoritarian behaviour) and failure to take advantage of objectively available offers. This happens, for example, out of shame at being admitted to a psychiatric ward‘ (p. 137). | JBI level 3, 100% MMAT |
|  | ‘Social reasons for admission should be viewed in the context of medical and nursing reasons‘ (p.137). | JBI level 3, 100% MMAT |
|  | ‘Complex needs arise from the presence of dementia. These needs and their severity, as well as the social environment, determine when a system “breaks down”, “no longer works” and referrals become necessary‘ (p. 138). | JBI level 3, 100% MMAT |
|  | ‘The personalities of the individuals involved are an important factor. Caregivers decide on care settings themselves, based on their personal experiences and preferences‘ (p. 138). | JBI level 3, 100% MMAT |
|  | ‘This means that hospital stays, especially in geriatric psychiatry, can also have advantages. In crisis and transitional situations, this period offers an opportunity for decision-making and/or switching to a different care concept, among other things, and is also actively used for this purpose‘ (p. 151). | JBI level 3, 100% MMAT |
|  | ‘The strain on the support system and its limitations are also evident in the lack of suitable care facilities, such as nursing homes for people with severe dementia and a need for physical activity. Even institutions with very good care concepts refuse to admit them, forcing families to search for nursing homes outside their region‘ (p. 142). | JBI level 3, 100% MMAT |
|  | ‘Added to this are, for example, bed shortages in geriatric psychiatry with longer waiting times and/or multiple internal transfers‘ (p. 142). | JBI level 3, 100% MMAT |
| **Study/article** | **Qualitative findings** | **Level of evidence/ quality appraisal score** |
| **Pöschel & Spannhorst, 2018a,** qualitative part of mixed-method study (expert interviews) | ‘When considering the overall social, medical and nursing care needs, behavioural disorders are the central finding and the central medical and nursing diagnosis. They occur in various combinations and forms, fluctuate greatly *and appear to have the greatest influence on the social, nursing and medical care of people with dementia*‘ (p. 159) | JBI level 3, 100% MMAT |
|  | ‘A wide range of behavioural disorders that arise in all [...] known care settings, necessitate a change in treatment and often result in a change in the care setting‘ (p. 159). | JBI level 3, 100% MMAT |
|  | ‘Behavioural disorders such as undressing, smearing faeces on walls, reversal of the day-night rhythm, wandering tendencies and aggression towards other residents lead to decompensation not only in the home environment but also in professional care settings‘ (p.159) | JBI level 3, 100% MMAT |
|  | ‘Assessments of the necessity of admission and the options for care have changed and will continue to change. *„We don't refer everyone. We also have lots of crazy people who we don't refer, but who aren't suffering terribly, subjectively speaking, and who are able to function reasonably well in everyday life. You wouldn't refer them just because they're a bit crazy, you wouldn't do that. Maybe people did that in the past, but they don't do it anymore.“‘* (p. 159) | JBI level 3, 100% MMAT |
|  | ‘However, there are also expectations among the actors themselves to rely on each other's assessments in order to achieve good care. If this does not succeed, it is perceived as disruptive‘ (p. 143). | JBI level 3, 100% MMAT |
|  | ‘[...] because gaps repeatedly occur in formal communication, which can then lead to interruptions in care for those affected, as the lead times for the professional actors are then insufficient‘ (p. 149). | JBI level 3, 100% MMAT |
|  | ‘This section addresses the problem of transferring one's own standards when assessing risk potential in care settings, as well as the associated insight that contexts should be evaluated not only on the surface, but also in light of the different ‘role’ behaviour of the actors involved. Added to this is the problem that, from the external perspective of the ‘professional’, it is difficult to assess the actual extent of the support required‘ (p. 150). | JBI level 3, 100% MMAT |
|  | ‘This leads to a situation where, even against one's better judgement, living and care situations must be accepted until they become unbearable and collapse. *„So, you always have to respect that your own standards are completely irrelevant. ... even in situations in human interaction where role behaviour simply has a different functionality than we are used to“‘* (p. 150) | JBI level 3, 100% MMAT |
|  | ‘GPs describe the characteristics of conditions that often lead to inpatient referral of people with dementia: These are primarily symptoms that are masked by behavioural disorders/delirium or cannot be classified with certainty, an unclear dynamic development and the indispensability of technical aids for diagnosis and therapy that are only available in an inpatient setting (e.g. X-ray examination or intravenous antibiotic therapy)‘ (p. 153). | JBI level 3, 100% MMAT |
| **Study/article** | **Qualitative findings** | **Level of evidence/ quality appraisal score** |
| **Pöschel & Spannhorst, 2018a,** qualitative part of mixed-method study (expert interviews) | ‘The legal guardian emphasises the legitimacy of admissions on medical grounds when increased support or assistance is required: [...] *„What I sometimes encounter is that institutions also contact us and say ‚we are no longer able to provide the necessary care [...]‘ I think that, as a rule, this is done through a doctor and is then justified on medical grounds*.“‘ (p. 154) | JBI level 3, 100% MMAT |
| **Pöschel & Spannhorst, 2018a,** qualitative part of mixed-method study (group discussion) | ‘They [GPs] are quite willing to work through their own checklist prior to admission, which could be drawn up in advance together with the geriatric psychiatry department and could address common issues such as desirable preliminary examinations prior to inpatient admission. This would enable conditions that cause restlessness in patients, such as infections, to be identified and treated on an outpatient basis more often, so that admission might still be avoided‘ (p. 119). | JBI level 3, 60% MMAT |
|  | ‘A planned and undoubtedly sensible referral of subacute cases is almost never possible due to the shortage of beds; this is only possible when cases become acute‘ (p. 119). | JBI level 3, 60% MMAT |
|  | ‘With regard to the interface at the time of discharge from inpatient treatment, reliable and timely communication between hospital doctors and general practitioners is essential in order to avoid inadequate care and risks.[...] However, it would be desirable to receive information before the day of discharge, especially if a new GP is involved [...] which is usually the case for discharges to care homes if the patient did not live there previously‘ (p. 119). | JBI level 3, 60% MMAT |
| **Van Voorden et al., 2024,**  qualitative part of mixed-method study (interviews) | ‘At admission, the vast majority of patients used many different types of psychotropic drugs,  often without a good rationale, according to the interviewees.‘ (p. 5) | JBI level 3, 100% MMAT |
|  | ‘Some interviewees mentioned that the severe challenging behaviour for which patients were admitted was not present after admission in a few cases and suggested that another social and/or physical environment may explain this.‘ (p. 5) | JBI level 3, 100% MMAT |
|  | ‘Tolerance of a certain level of challenging behaviour was essential in this phase to enable effective observation. [...] “*If someone wants it [the challenging behaviour] gone immediately, it changes your perspective. There’s a certain peace like: ‘okay, this is it, let’s see where we still can be of any help to someone“’* (p. 7) | JBI level 3, 100% MMAT |
|  | ‘Prior to admission, it was considered critically whether treatment was needed. Units considered which interventions had been used to date and often gave advice to prevent admission. In one unit, the interviewee mentioned they insisted on consultation in the current residence beforehand, thereby preventing about one-third of proposed admissions. This prior consultation was conducted by the physician responsible for medical care or the psychologist, sometimes together with a nursing staff member.‘ (p. 7) | JBI level 3, 100% MMAT |
| **Study/article** | **Qualitative findings** | **Level of evidence/ quality appraisal score** |
| **Van Voorden et al., 2024,**  qualitative part of mixed-method study (interviews) | ‘Although interviewees strived to taper off the psychotropic drugs, they did not always consider  this to be possible. They were satisfied when they could reduce the number of different types of psychotropic drugs and prescribe psychotropic drugs with a better rationale. Overall, interviewees mentioned that guidelines held limited usefulness for the treatment in these  Units. „Almost everything we do is no longer evidence-based and that’s a huge problem.“ „We all have mainly expert opinions, meaning the knowledge of people who know more about it“‘ (p. 7) | JBI level 3, 100% MMAT |
|  | ‘Some interviewees mentioned that discharge seemed to be impossible for some patients, sometimes after a probation discharge: *“I might say that we go on trying, but that’s actually not always the case. Because at a certain moment we simply don’t know any more, than it’s manageable for the unit.” “Exactly, sometimes it’s manageable for us, and then we say that this is the best possible. But we mean that it’s not manageable in a regular unit”*‘.  ‘Some units strongly invested in discharge by inviting the nursing staff of the proposed unit for discharge to care for the patient together to explain behavioural guidance in practice‘ (p. 7). | JBI level 3, 100% MMAT |

Table S5 Presentation of interpretative or opinion-based quotations, evidence level and quality appraisal score

| **Study/articles** | **Interpretative or opinion-based findings (quotations)** | **Level of evidence/ quality appraisal score** |
| --- | --- | --- |
| **Backhouse et al., 2018,**  systematic review, (extracted from the consolidation of the results) | **‘There was some evidence that crises are not being resolved long-term**, (since seven studies reported the same patients being re-admitted within the study timescale)‘ (p. 109) | JBI level 1, 100% JBI systematic review tool, but author’s interpretation |
|  | ‘There is little good quality evidence showing how best to manage behavioural crises in dementia. Interventions employed to resolve crises were often not clearly specified‘ (p. 109). | JBI level 1, 100% JBI systematic review tool, but author’s interpretation |
|  | ‘From the information reported we could not determine whether behavioural crises occurred due to facility and/or caregiver variables or person with dementia variables‘ (p. 109). | JBI level 1, 100% JBI systematic review tool, but author’s interpretation |
| **Study/articles** | **Interpretative or opinion-based findings (quotations)** | **Level of evidence/ quality appraisal score** |
| **Pöschel & Spannhorst, 2018a,** (extracted from the quantitative part, document analysis) | ‘Readmissions of patients who had already been treated at least once in a geriatric psychiatric ward were considered an indication of particularly intensive treatment needs or repeatedly decompensated outpatient situations‘ (p. 101).  (based on: ‘Of the 100 cases, 26 were admitted to the department at least twice (so-called readmissions‘) (p. 101). | JBI level 4, 60% MMAT, author‘s interpretation |
| **Pöschel & Spannhorst, 2018a,** (extracted from the consolidation of the results of the entire mixed method study) | ‘However, the resilience of the respective environment was subject to considerable variation. What was tolerable and compensable for one support system led to escalation and decompensation in another setting‘ (p. 170-171). | JBI level 4, 80% MMAT, mixed method study, but author’s interpretation |
|  | ‘A demonstrable significant increase in behavioural disorders. [...] According to expert opinion and based on the documentation, these are the most common reasons for decompensation in a caring environment and justify the need for admission‘ (p. 171). | JBI level 4, 80% MMAT, mixed method study, but author’s aggregation and interpretation |
|  | ‘Despite the tendency towards a drastic improvement in symptoms in the majority of patients at the end of their hospital stay, a number of behavioural disorders persisted‘ (p. 171). | JBI level 4, 80% MMAT, mixed method study, but author’s interpretation |
|  | ‘Somatic diseases therefore often constituted a factor accompanying or guiding treatment‘ (p. 172). | JBI level 4, 80% MMAT, mixed method study, but author’s interpretation |
| **Pöschel & Spannhorst, 2018a,** (extracted from the consolidation of the results of the entire mixed method study) | ‘According to the experts, decisions to refer patients to hospital depended largely on the current care setting and not solely on the presence of a disease‘ (p. 172) | JBI level 4, 80% MMAT, mixed method study, but author’s aggregation) |
|  | These three areas – social, medical and nursing care – interact to reflect the complexity of the needs that are sensitive to lifestyle and how they interact. A change in one area immediately necessitates adjustments in other areas. If this is not possible, an imbalance or decompensation occurs, which manifests itself as an escalation or crisis (p. 174) | JBI level 4, 80% MMAT, mixed method study, but author’s interpretation |
| **Study/articles** | **Interpretative or opinion-based findings (quotations)** | **Level of evidence/ quality appraisal score** |
| **Pöschel et al., 2018,** (extracted from the consolidation of the results of the mixed method study) | ‘The study points to complex needs among people with dementia at the interface between outpatient and inpatient care. Often, a combination of social, nursing and medical reasons, to varying degrees, are found to be the cause of inpatient admission. The authors summarise these multidimensional needs under the term DCSD (Dementia Care Sensitive Demands) in order to specifically illustrate the specific complexity of the overall situation of people with dementia‘ (p. 155) | JBI level 4, 80% MMAT, mixed method study, but author’s aggregation |
|  | ‘Delusions, fears, defensive behaviour and motor restlessness place considerable strain on nursing staff and are key factors in the decision to refer a patient to hospital‘ (p. 155) | JBI level 4, 80% MMAT, mixed method study, but author’s aggregation |
|  | ‘Social reasons, such as conflict situations, are only mentioned once as an explicit reason for admission, despite significantly more frequent mentions of aggression towards objects or persons‘ (p. 155) | JBI level 4, 80% MMAT, mixed method study, but author’s aggregation |
|  | ‘Combining the three characteristics mentioned above [chronic and acute somatic illness, unstable support system, increased need for care], challenging behaviour (BPSD) appears to be the culmination of multidimensional problems in DCSD‘ (p. 156) | JBI level 4, 80% MMAT, mixed method study, but author’s interpretation |
|  | ‘It is important to note that despite the highly specialised focus of the Department of Geriatric Psychiatry, although delirium was successfully treated in all cases documented here, behavioural disorders were not successfully treated‘ (p. 156). | JBI level 4, 80% MMAT, mixed method study, but author’s interpretation |
| **Wetterling, 2015**, (extracted from the consolidation of the results) | ‘The significant number of cases in which insufficient food intake was only detected by the admitting physicians or could only be observed during inpatient treatment shows that food intake depends on the environment‘ (p. 45). | JBI level 3, 60% MMAT, mixed method study, but author’s interpretation |
|  | ‘In addition to the problems residents have in adjusting to the routines there, other difficulties such as limited attention due to tight schedules for nursing staff should also be mentioned‘ (p. 45). | JBI level 3, 60% MMAT, mixed method study, but author’s interpretation |
| **Spannhorst et al., 2020,** practice report, categorised as opinion paper | The hospital setting can be an artificially shielded environment – stabilisation measures achieved there often cannot be directly transferred to the home environment (p. 713). | JBI level 5, 100% JBI-Checklist for Textual Evidence: Expert Opinion |
|  | ‘There is still a glaring shortage of nursing staff in nursing homes. As a result, recommendations such as one-to-one care, empathetic listening and assisted mobilisation of restless patients with dementia are in line with guidelines [S-3 Dementia Guideline], but often impossible to implement‘ (p. 716). | JBI level 5, 100% JBI-Checklist for Textual Evidence: Expert Opinion |
| **Study/articles** | **Interpretative or opinion-based findings (quotations)** | **Level of evidence/ quality appraisal score** |
| **Spannhorst et al., 2020,** practice report, categorised as opinion paper | ‘Even in cases of aggressive behaviour towards others on the part of residents, the desire for physical separation sometimes takes precedence over the desire to treat the behavioural disorder on site‘ (p. 718) | JBI level 5, 100% JBI-Checklist for Textual Evidence: Expert Opinion |
|  | ‘This overall situation [ban on visits during the COVID-19 pandemic, visits by StäB employees also suspended] inevitably led to increased pressure to admit patients to inpatient geriatric psychiatric care‘ (p. 718) | JBI level 5, 100% JBI-Checklist for Textual Evidence: Expert Opinion |
| **Richler et al., 2023,** unsystematic review, case analysis, categorised as opinion paper | ‚The absence of plans for BPSD management resulted in emotional distress among the family, the treatment team, and the patient‘ (p. 22) | JBI level 5, 100% JBI-Checklist for Textual Evidence: Expert Opinion |
|  | ‘It [Alzheimer’s Association Dementia Care Practice Recommendations] does state that there are no FDA-approved medications for the treatment of BPSD‘ (p. 25) | JBI level 5, 100% JBI-Checklist for Textual Evidence: Expert Opinion |
| **Richler et al., 2023, ,** unsystematic review, case analysis (extracted from the consolidation, interpretation of the results) | ‘Providers are unsure of how to engage in discussions around BPSD due to lack of knowledge, lack of expertise, and difficulty broaching such an uncomfortable topic with patients and families. Avoiding such discussions, however, does not change the fact that BPSD is common, complex, and can be clinically, logistically, and emotionally demanding to manage‘ (p. 25) | JBI level 5, 100% JBI-Checklist for Textual Evidence: Expert Opinion |
|  | ‘The family’s understanding of the disease course and care options was limited and further complicated by unrealistic beliefs and insufficient information. Complex decisions had to be made expeditiously, leading to discomfort, shame, and embarrassment among family as well as providers‘ (p. 25) | JBI level 5, 100% JBI-Checklist for Textual Evidence: Expert Opinion |
|  | ‘The lack of understanding of dementia as a progressive disease may serve as a barrier for the family to have realistic views of the patient’s prognosis and treatment options‘ (p. 25) | JBI level 5, 100% JBI-Checklist for Textual Evidence: Expert Opinion |
|  | ‘BPSD is extremely common among people living with dementia, yet most patients and family members have minimal or no knowledge of the fact that BPSD is part of dementia‘ (p. 25) | JBI level 5, 100% JBI-Checklist for Textual Evidence: Expert Opinion |
| **Study/articles** | **Interpretative or opinion-based findings (quotations)** | **Level of evidence/ quality appraisal score** |
| **Richler et al., 2023,** (extracted from the consolidation, interpretation of the results) | ‘There is no FDA-approved treatment for BPSD. Perhaps the most distressing aspect of BPSD for the patient and their family is that there is no cure and no FDA-approved treatment for BPSD. Families have certain expectations for clinical outcomes based on a lack of understanding of treatment options for BPSD. They are frequently unaware that they will likely have to make complex medical decisions around BPSD management, including the use of antipsychotic medications, which carry a black box warning for increasing mortality when used in people living with dementia‘ (p. 26) | JBI level 5, 100% JBI-Checklist for Textual Evidence: Expert Opinion |
|  | ‘Placement of patients with dementia who are not safe in the community into appropriate care facilities can be complicated. For those with BPSD, the challenge can be even more pronounced. Problematic behaviours associated with BPSD can interfere with care and cause disruption in the facility. The availability of memory care units that can handle such behaviours is limited, and resources have become even more scarce due to the COVID-19 pandemic. Management of BPSD for placement into these facilities is further complicated by the necessity of achieving a balance between agitation and sedation.‘ (p. 26) | JBI level 5, 100% JBI-Checklist for Textual Evidence: Expert Opinion |
|  | All too often, the conversations around BPSD do not occur until the patient with dementia is admitted to a geriatric psychiatry unit. However, an inpatient psychiatric unit does not provide the platform suitable for the providers and the family to have ACP [Advance Care Planning] in the most comprehensive way (p. 26) | JBI level 5, 100% JBI-Checklist for Textual Evidence: Expert Opinion |
| **Pöschel & Spannhorst, 2018b,** secondary data analysis, categorised as opinion paper | ‘The three areas – social, medical and nursing – interact to reflect the complexity of the needs that are sensitive to lifestyle and how they interact. A change in one area immediately necessitates adjustments in other areas. If this is not possible, an imbalance or decompensation occurs, which manifests itself as an escalation or crisis, especially at the interface between outpatient and inpatient care‘ (p. 51) | JBI level 5, 100% JBI-Checklist for Textual Evidence: Expert Opinion |
|  | ‘This concept [DCSD], similar to combining different factors into a syndrome, [...] serves to describe, analyse, present and better understand the dynamics and complexity of dementia. The needs described can come to the fore or recede into the background with varying intensity‘ (p. 51) | JBI level 5, 100% JBI-Checklist for Textual Evidence: Expert Opinion |
|  | ‘Decompensation occurs when it is not possible to find common goals, maintain reliable communication or when cooperation is called into question due to a lack of human or time resources‘ (p. 51-52) | JBI level 5, 100% JBI-Checklist for Textual Evidence: Expert Opinion |
| **Study/articles** | **Interpretative or opinion-based findings (quotations)** | **Level of evidence/ quality appraisal score** |
| **Pöschel & Spannhorst, 2018b,** secondary data analysis, categorised as opinion paper | ‘Individual perceptions of stressful situations can be linked to psychosocial factors. These psychological phenomena, which influence the current behaviour of individuals, must be understood in the context of personal relationship and role experiences and each individual's own biography. These experiences influence behaviour, symptom severity, motivation to provide care and support, and actual and perceived stress. This applies both to people with dementia and to their social environment‘ (p. 53) | JBI level 5, 100% JBI-Checklist for Textual Evidence: Expert Opinion |
|  | ‘Communication takes place on various levels. It is therefore perceived differently and can lead to misunderstandings. This happens in particular when the person with dementia is out of sight, their communication skills change, and people communicate over their head‘ (p. 53). | JBI level 5, 100% JBI-Checklist for Textual Evidence: Expert Opinion |
|  | ‘The urgency of admissions depends on the severity of the challenging behaviours, the potential for violence and aggression, and the capacity of the care setting‘ (p. 54). | JBI level 5, 100% JBI-Checklist for Textual Evidence: Expert Opinion |
|  | ‘The explosive increase in challenging behaviours at the time of admission clearly shows that these behaviours in particular contribute significantly to the decompensation of the current care situation‘ (p. 54) | JBI level 5, 100% JBI-Checklist for Textual Evidence: Expert Opinion |
|  | ‘A significant proportion of behavioural problems persist even after an acute phase of illness‘ (p. 54) | JBI level 5, 100% JBI-Checklist for Textual Evidence: Expert Opinion |
