# Supplementary material for: From Nursing Homes to Geriatric Psychiatry: Contextual Factors Associated With the Admission of People With Dementia and Behaviour That Challenges—An Integrative Review
Source: Nurs Open. 2026 May 16;13(5):e70592. doi: 10.1002/nop2.70592 (PMC13179823; doi:10.1002/nop2.70592)
Supplement: Supplementary file 3 — Table S6: Coding table with the theme, sub‐themes, categories, codes, key quotes and the respective sources. [file NOP2-13-e70592-s002.docx]

Table S6 Coding table with the theme, sub-themes, categories, codes, key quotes and the respective sources

| **MAIN THEME: CRISIS-DETERMINING FACTORS** | | | | |
| --- | --- | --- | --- | --- |
| **Sub-theme** | **Category** | **Codes** | **Key quotes** | **Source** |
| **Multifactoriality and dimensionality of dementia and behaviour that challenges** | Higher care requirements and decompensating situations | Complex care needs due to social, nursing and medical reasons; chronic and acute somatic illnesses, unstable support system and increased care needs lead to behaviour that challenges; interaction between demands and necessary adjustments; immense burden for nursing staff; behaviour that challenges (especially certain types of behaviour) lead to decompensation and crisis situation | ‘The study points to complex needs among people with dementia at the interface between outpatient and inpatient care. Often, a combination of social, nursing and medical reasons, to varying degrees, are found to be the cause of inpatient admission. The authors summarise these multidimensional needs under the term DCSD (Dementia Care Sensitive Demands) in order to specifically illustrate the specific complexity of the overall situation of people with dementia’ (Pöschel et al., 2018, p. 155)  ‘These three areas – social, medical and nursing care – interact to reflect the complexity of the needs that are sensitive to lifestyle and how they interact. A change in one area immediately necessitates adjustments in other areas. If this is not possible, an imbalance or decompensation occurs, which manifests itself as an escalation or crisis’ (Pöschel & Spannhorst 2018a, p. 174)  Agitation and aggression were described in all included studies as key triggers for crisis situations (Backhouse et al. 2018, p. 102). | Pöschel & Spannhorst, 2018a; Pöschel & Spannhorst, 2018b; Pöschel et al., 2018; Spannhorst et al., 2020; Backhouse et al., 2018 |
|  | Complex causes and complex treatment orders | Behaviour that challenges as the tip of multidimensional problems; somatic and psychiatric comorbidities influence behaviour that challenges and treatment; social conflicts can lead to admission; social and physical environment in particular influences the occurrence of behaviour that challenges | ‘The occurrence of chronic and acute somatic illnesses, an unstable support system and increased care needs in combination appear to lead to challenging behaviour in DCSD as the tip of multidimensional problems’ (Pöschel et al., 2018, p. 156)  ‘Somatic diseases therefore often constituted a factor accompanying or guiding treatment’ (Pöschel & Spannhorst 2018a, p. 172).  ‘The most common diagnosis on admission was challenging behaviour, accounting for half of cases, followed by delirium and confusion in about a quarter of cases, [...], followed by a smaller proportion of cases with the symptom complex ‘psychotic symptoms, delusions or misidentification of persons.’ (Pöschel & Spannhorst, 2018a, p. 98)  ‘Social reasons, such as conflict situations, are only mentioned once as an explicit reason for admission, despite significantly more frequent mentions of aggression towards objects or persons’ (Pöschel et al., 2018, p. 155) | Pöschel & Spannhorst 2018a; Pöschel et al., 2018; van Voorden et al., 2024; Wetterling, 2015 |
| **Sub-theme** | **Category** | **Codes** | **Key quotes** | **Source** |
| **Multifactoriality and dimensionality of dementia and behaviour that challenges** | Limited effective treatment options | Behaviour that challenges occurs in various combination and forms; onset of behaviour that challenges is unclear; little high-quality evidence for management of behaviour that challenges, therefore no long-term solution; despite high specialisation in geriatric psychiatry no successful treatment of behaviour that challenges; clinical condition of patients deteriorates due to behaviour that challenges; no sufficiently effective interventions for treatment (including FDA-approved drugs); limited understanding of the course of the disease and the available treatment options of relatives; GPs feel helpless with dementia patients; unnecessary use of psychotropic drugs; treatment is based more on expert opinion than on guidelines; Combination of non-pharmacological and pharmacological measures more commonly used than purely pharmacological measures; stabilisation measures from geriatric psychiatry not directly transferable | ‘It is important to note that despite the highly specialised focus of the Department of Geriatric Psychiatry, although delirium was successfully treated in all cases documented here, behavioural disorders were not successfully treated‘ (Pöschel et al., 2018, p. 156).  ‘There was some evidence that crises are not being resolved long-term, (since seven studies reported the same patients being re-admitted within the study timescale)‘ (Backhouse et al., 2018, p. 109).  It [Alzheimer’s Association Dementia Care Practice Recommendations] does state that there are no FDA-approved medications for the treatment of BPSD (Richler et al., 2023, p. 25).  About half of general practitioners agreed that they some-times feel helpless in their relationship with their patients suffering from dementia (Pöschel & Spannhorst, 2018a, p. 115).  ‘Although interviewees strived to taper off the psychotropic drugs, they did not always consider this to be possible. They were satisfied when they could reduce the number of different types of psychotropic drugs and prescribe psychotropic drugs with a better rationale. Overall, interviewees mentioned that guidelines held limited usefulness for the treatment in these Units. *„Almost everything we do is no longer evidence-based and that’s a huge problem.“ „We all have mainly expert opinions, meaning the knowledge of people who know more about it“‘* (van Voorden et al., 2024, p. 7). | Backhouse et al., 2018; Pöschel & Spannhorst, 2018a; Pöschel & Spannhorst, 2018b; Pöschel et al., 2018; Richler et al., 2023; Spannhorst et al., 2020; van Voorden et al., 2024; Wetterling, 2015 |
| **Sub-theme** | **Category** | **Codes** | **Key quotes** | **Source** |
| **Structural and systemic barriers** | Care deficit due to lack of resources | Limited attention from nursing staff due to low staffing levels and time constraints; guideline-oriented approach not feasible due to lack of personnel; shortage of beds in geriatric psychiatry and long waiting times until admission; planned transfers of subacute cases to geriatric psychiatry not possible, only when acute; lack of specialised care facilities; long waiting times/supraregional search for a nursing home place necessary; resources even scarcer during the COVID-19 pandemic; difficult identification of symptoms masked by behavioural disorders/delirium due to a lack of technical aids. | ‘In addition to the problems residents have in adjusting to the routines there, other difficulties such as limited attention due to tight schedules for nursing staff should also be mentioned‘ (Wetterling, 2015, p. 45).  ‘There is still a glaring shortage of nursing staff in nursing homes. As a result, recommendations such as one-to-one care, empathetic listening and assisted mobilisation of restless patients with dementia are in line with guidelines [S-3 Dementia Guideline], but often impossible to implement‘ (Spannhorst, 2020, p. 716).  ‘Added to this are, for example, bed shortages in geriatric psychiatry with longer waiting times and/or multiple internal transfers‘ (Pöschel & Spannhorst, 2018a, p. 142).  ‘GPs describe the characteristics of conditions that often lead to inpatient referral of people with dementia: These are primarily symptoms that are masked by behavioural disorders/delirium or cannot be classified with certainty, an unclear dynamic development and the indispensability of technical aids for diagnosis and therapy that are only available in an inpatient setting (e.g. X-ray examination or intravenous antibiotic therapy)‘ (Pöschel & Spannhorst, 2018a, p. 153). | Pöschel & Spannhorst, 2018a; Richler et al., 2023; Spannhorst et al., 2020; Wetterling, 2015; |
|  | Capacity limit of the care system | Decisions about referring patients to hospital depend on current care conditions; social environment determines when the system fails; time of admission/ discharge depends on the place of origin; different resilience in the respective care contexts; psychosocial factors can influence behaviour, symptom severity, motivation to provide care, and burdens of those involved; medical justification of social reasons for admission; increased admission pressure in geriatric psychiatric clinics due to visitor restrictions during the COVID-19 pandemic | ‘According to the experts, decisions to refer patients to hospital depended largely on the current care setting and not solely on the presence of a disease‘ (Pöschel & Spannhorst, 2018a, p. 172)  ‘In accordance with the guidelines for access to services, this [a socially indicated referral] is actively used in the context of referrals, through legitimate diagnoses, to initiate services such as clinical treatment‘ (Pöschel & Spannhorst, 2018a, p. 134).  ‘This overall situation [ban on visits during the COVID-19 pandemic, visits by StäB employees also suspended] inevitably led to increased pressure to admit patients to inpatient geriatric psychiatric care‘ (Spannhorst et al., 2020, p. 718) | Pöschel & Spannhorst, 2018a; Pöschel & Spannhorst, 2018b; Spannhorst et al., 2020; van Voorden et al., 2024 |
| **Sub-theme** | **Category** | **Codes** | **Key quotes** | **Source** |
| **Lack of effective interprofessional and patient-centred collaboration** | Communication deficits | Lack of communication as a main reason for poor collaboration; lack of knowledge about each other's working methods; lack of suitable checklists to identify illnesses that lead to behaviour that challenges; interruption of care due to poor formal communication; side effects during treatment; late discussions about behaviour that challenges with relatives; cross-sector communication (assessment of the necessity of admission, recommendations to avoid admission) | ‘With regard to the interface at the time of discharge from inpatient treatment, reliable and timely communication between hospital doctors and general practitioners is essential in order to avoid inadequate care and risks.[...] However, it would be desirable to receive information before the day of discharge, especially if a new GP is involved [...] which is usually the case for discharges to care homes if the patient did not live there previously‘ (Pöschel & Spannhorst, 2018a, p. 119).  ‘Experts cite poor communication as a key factor hindering cooperation. If information is required but not available, an unnecessary amount of resources must be spent on research‘ (Pöschel & Spannhorst, 2018a, p. 132).  ‘Communication takes place on various levels. It is therefore perceived differently and can lead to misunderstandings. This happens in particular when the person with dementia is out of sight, their communication skills change and people communicate over their head‘ (Pöschel & Spannhorst, 2018b, p. 53). | Pöschel & Spannhorst, 2018a; Pöschel & Spannhorst, 2018b; Richler et al., 2023; van Voorden et al., 2024 |
|  | Differences in views, evaluation criteria, and practices among stakeholders | Lack of acceptance of possible solutions; lack of reliance on professional judgements; lost holistic perspective; actors' own standards for assessing patient risks difficult to transfer; consideration of actor's role behaviour; non-compliance with agreements; different views and unrealistic expectations regarding treatment (relatives/professionals); non-use of objectively available services due to shame of relatives; lack of knowledge about options in regular nursing homes; acceptance of life and care situations until escalation | ‘Further problems can arise from the tunnel vision of the actors themselves, which leads to a loss of holistic perspective‘ (Pöschel & Spannhorst, 2018a, p. 133).  ‘The family’s understanding of the disease course and care options was limited and further complicated by unrealistic beliefs and insufficient information. Complex decisions had to be made expeditiously, leading to discomfort, shame, and embarrassment among family as well as providers‘ (Richler et al., 2023, p. 25)  ‘Some interviewees mentioned that discharge seemed to be impossible for some patients, […]: *“I might say that we go on trying, but that’s actually not always the case. Because at a certain moment we simply don’t know any more, than it’s manageable for the unit.” “Exactly, sometimes it’s manageable for us, and then we say that this is the best possible. But we mean that it’s not manageable in a regular unit”‘* (van Voorden et al., 2024, p. 7). | Pöschel & Spannhorst, 2018a; Richler et al., 2023; van Voorden et al., 2024; |
